# Stathmin Serine 16 Phosphorylation Is a Key Regulator of Cell Cycle Progression Without Activating Migration and Invasion In Vitro

**DOI:** 10.3390/cancers17142322

**Published:** 2025-07-12

**Authors:** Paul L. Deford, Andrew P. VonHandorf, Brian G. Hunt, Simran Venkatraman, Susan E. Waltz, Katherine A. Burns, Susan Kasper

**Affiliations:** 1 Kettering Laboratory, Department of Environmental and Public Health Sciences, University of Cincinnati College of Medicine, 160 Panzeca Way, Cincinnati, OH 45267-0056, USA; paul.deford@corteva.com (P.L.D.); venkask@ucmail.uc.edu (S.V.); burns2ki@ucmail.uc.edu (K.A.B.); 2 Center for Autoimmune Genomics and Etiology, Cincinnati Children’s Hospital Medical Center, 3333 Burnet Avenue, Cincinnati, OH 45229-3039, USA; andrew.vonhandorf@cchmc.org; 3 Department of Cancer Biology, Vontz Center for Molecular Studies, University of Cincinnati College of Medicine, 3125 Eden Avenue, P.O. Box 670521, Cincinnati, OH 45267-0521, USA; brian.hunt@yale.edu (B.G.H.); waltzse@ucmail.uc.edu (S.E.W.)

**Keywords:** Stathmin 1 (STMN1), phosphorylation, serine 16 (S16), hepatocyte growth factor/scatter factor (HGF/SF), cell cycle, prostate cancer, breast cancer, metastasis

## Abstract

**Background**: Treatment of metastatic cancer remains a challenge, because cancer cells acquire resistance even to the most contemporary therapies. This study analyzed the role of the phosphoprotein Stathmin 1 (STMN1) in regulating cancer cell growth and metastatic potential. **Methods**: Public datasets with metastatic castration-resistant prostate cancer (mCRPC) and breast cancer (BC) were analyzed to determine the interrelationship between STMN1, hepatocyte growth factor (HGF) and MET proto-oncogene (MET) expression, overall survival, and response to chemotherapy. Site-directed mutagenesis, cell cycle analysis, proliferation, and migration and invasion assays determined the impact of STMN1 phosphorylation on proliferation and metastatic potential. **Results**: Increased STMN1 associates with HGF and MET gene expression in mCRPC, and taxane chemotherapy further increases HGF expression. STMN1 and HGF are highest, and overall survival is poorest in mCRPC in the liver compared to other sites, implying the metastatic site influences their expression levels and potentially the pattern of metastatic spread. Increased STMN1 and MET also predict taxane responsiveness in BC patients. Analysis of STMN1 serine (S)16, 25, 38, and 63 determined that total (t) STMN1 and STMN1 S16 phosphorylation (pSTMN1^S16^) are co-regulated by HGF/MET during cell cycle progression, pSTMN1^S16^ alone can promote cell proliferation, and pSTMN1^S16^ shortens the cell cycle similar to HGF treatment, while STMN1^S16^ dephosphorylation lengthens the cell cycle to arrest cell growth in G2/M, similar to HGF plus the MET inhibitor AMG337. Importantly, STMN1^S16^ does not promote metastasis. **Conclusions**: Selectively inhibiting STMN1^S16^ phosphorylation may provide an alternative strategy for inhibiting MET-mediated cell growth to eliminate metastatic cancer cells and inhibit further metastasis.

## Simple Summary

Treating metastatic prostate cancer is challenging because it will become resistant to most forms of treatment. This study investigated Stathmin 1, a protein that regulates cancer cell growth, and found that Stathmin 1 levels were high in metastatic breast and prostate cancer. High Stathmin 1 expression was also associated with poor overall survival, and survival worsened when prostate cancer metastasized to the liver compared to other organs. In cell culture assays, the addition of a phosphate molecule to serine 16 on the Stathmin 1 protein increased cancer cell growth, while removing the phosphate inhibited cell growth. Notably, Stathmin 1 phosphorylated on serine 16 did not promote metastasis. Thus, selectively preventing Stathmin 1 serine 16 phosphorylation may provide an alternative strategy for inhibiting growth factor-mediated metastatic cell growth in combination with current therapies used to eliminate metastatic cancer cells while preventing/inhibiting further metastasis.

## 1. Introduction

For all of the advances made in the treatment of cancer, one statistic remains unchanged—most deaths from solid tumors are caused by metastasis. The aggressive, systemic treatment of metastatic cancers like prostate and breast cancer consists of multiple approaches, including hormonal therapy, chemotherapy, targeted therapies, and/or immunotherapy [1,2]. Recent work on the role of the cell cycle in cancer growth provides evidence that, instead of undergoing uncontrolled cell division, cancer cells are primarily compromised in their ability to exit the cell cycle and therefore continue to divide [3]. Taxane-based chemotherapies target microtubules during mitosis and interphase, causing stabilization of the mitotic spindle, leading to arrest in mitosis and ultimately cancer cell death [4]. While taxanes showed an improvement in overall survival in metastatic (m) cancer, they are not curative [4,5]. For example, Docetaxel (DTX), a standard first-line chemotherapy for patients with metastatic castration-resistant prostate cancer (mCRPC), binds to β-tubulin to promote microtubule assembly and stabilization of the mitotic spindle, resulting in G2/M arrest, apoptosis, and cell death [6,7]. While DTX treatment extends overall patient survival, mCRPC still develops drug resistance [6,7].

The phosphoprotein Stathmin (STMN1) is a relay protein that transmits upstream signals from multiple extracellular regulators such as ion channels, growth factors, hormones, and neurotransmitters via diverse second messenger pathways to regulate spindle formation during mitosis and microtubule formation during cell motility (reviewed in [8]). The complexity in STMN1 regulation is highlighted by four primary second messenger pathways, Calcium/Calmodulin-dependent Protein Kinase II (CAMKII), mitogen-activated protein kinases, p34 protein kinase/cyclin-dependent kinase 1 (p34^cdc2^/CDK1), and Protein Kinase A (PKA), which phosphorylate STMN1 serine (S) S16, S25, S38, and S63, respectively [9,10]. Phosphorylation inactivates STMN1, causing the release of α/β-tubulin dimers and making them available for spindle and microtubule formation [11]. STMN1 is reactivated through dephosphorylation to bind free α/β tubulin dimers to promote cell entry into a new interphase [12] or to cause the breakdown (a.k.a. as catastrophe) of microtubules [11]. Thus, cell proliferation and motility are hallmarks of STMN-mediated activity in the context of cancer development and progression.

STMN1 is overexpressed in many cancers, including prostate and breast cancer [8,13,14], and studies on STMN1 typically analyze total STMN1 expression by gene expression profiling and immunohistochemistry of clinical cancer samples or cancer cell lines. A limited number of these studies also include determining the levels of STMN1 phosphorylation of one or more of the four serines. In the only study addressing STMN1 phosphorylation in prostate cancer, Jurmeister et al. reported that the MELK kinase inhibitor OTS167 decreased S16 and S38 phosphorylation in C4-2b PCa cells, as determined by Phospho Explorer Antibody Arrays; however, the function of S16 and S38 phosphorylation in regulating C4-2b cell growth or metastasis was not addressed [15]. One study also investigated the effects of HGF on STMN1 phosphorylation in MDA-MB-231 human breast cancer cells and determined that HGF (50 ng/mL) promoted STMN1 phosphorylation at S25 and S38, which induced microtubule growth, STMN1 binding to the Pak1–WAVE2–kinesin complex via the selective phosphorylation of S38, and lamellipodia formation [16,17,18]. Thus, the study concluded that further clarification was required for determining the mechanisms by which the recruitment of phosphorylated STMN1 to this complex would induce membrane transport of the complex along microtubules to regulate cell migration and invasion through lamellipodia formation [16,17,18]. Taken together, these two studies indicate that our knowledge on the role of STMN1 phosphorylation in regulating PCa cell growth and metastasis at the cellular level is considerably understudied.

Other studies have interrogated the function of STMN1 phosphorylation at select serines in cancer cells other than PCa cells. For example, S16 phosphorylation was associated with paclitaxel-induced microtubule stability and apoptosis in acute lymphoblastic leukemia (ALL)-derived Jurkat and Namalwa cells [19,20]. Another study reported that the overexpression of the proapoptotic protein Siva1 promoted apoptosis, suppressed migration and invasion, and enhanced S16 phosphorylation in A2780 ovarian cells [19,20], with the implication that S16 phosphorylation regulated these processes in response to Siva1. In an immunohistochemical study, S38 phosphorylation was associated with poor overall survival in head and neck squamous cell carcinoma and endometrial cancer [21,22]. In studies investigating the phosphorylation of two STMN1 serines, Siva1 promoted S16 (but not S63) phosphorylation via calcium/calmodulin-dependent protein kinase II (CaMKII) in U2OS osteosarcoma cells [23]. In non-small cell lung cancer cells (NCI-H1299), downregulation of interleukin-10 (IL-10) expression downregulated STMN1 expression and its phosphorylation at S25 and S63 [24]. Interestingly, the knockdown of phosphatase of regenerating liver-3 (PRL-3) in K562 myeloid leukemia cells decreased total STMN1 expression and increased the phosphorylation of S16, S25, S38, and S63 [16,17,18]. In addition, PRL-3 silencing decreased proliferation, migration, and invasion, and therefore it was postulated that these PRL-3-mediated activities occurred in response to STMN1 phosphorylation [16,17,18]. The Epstein–Barr Virus kinase BGLF4 phosphorylated S16, S25, and S38 (but not S63) in nasopharyngeal carcinoma cells, and this observation implied that BGLF4 down-regulated STMN1 activity [16,17,18].

Taken together, these studies determined the levels of STMN1 phosphorylation in response to kinases and other regulatory factors. However, the function of STMN1 phosphorylation in regulating cyclins and other factors during cell cycle progression and proliferation were not investigated. Thus, the mechanisms by which the interplay between the four STMN1 phosphoserines regulate cell cycle progression, proliferation, and metastasis, and whether or not these activities are cancer cell type specific, remain to be established. Determining how the integration of STMN1 serine phosphorylation regulates cell growth and metastasis would provide much needed insight into why therapeutic strategies targeting cell division or microtubules in metastatic disease often fail.

The tumor microenvironment produces key regulators of cancer growth and metastasis, including growth factors such as hepatocyte growth factor (HGF), a growth factor that is produced by cancer-associated fibroblasts [25]. HGF is secreted into the tumor microenvironment, where it binds to the MET proto-oncogene/receptor tyrosine kinase receptor on cancer cells to activate MET signaling [25]. In DU-145 prostate cancer (PCa) cells, HGF is reported to increase proliferation; however, STMN1 phosphorylation is not evaluated in this landmark study (439 citations) [26]. Clinical studies report *MET* and *HGF* overexpression correlate with cancer progression and poor prognosis. In PCa, *MET* overexpression correlates with cancer progression [27,28], metastasis to bone and lymph nodes [29,30], and castration resistance [31]. Higher HGF plasma levels also correlate with metastatic PCa and decreased overall survival [32], and HGF expression is greater in PCa compared to benign prostate hyperplasia (BPH) [33]. Moreover, the increase in *HGF* is concomitant with increased *MET* expression [30]. Similarly, *MET* overexpression is observed in many other cancers, including breast cancer (BC) [34], where serum HGF levels increase with BC and are higher in patients with mBC [35]. Taken together, *STMN1*, *HGF*, and *MET* overexpression are associated with cancer progression. However, gaps in our knowledge remain on the interactions between these three factors in cancer progression, whether or not the metastatic site influences their expression and activity, and which STMN1 phosphoserines regulate PCa cell cycle progression, proliferation, and/or metastatic potential in response to HGF/MET signaling.

Since pSTMN1^S16^ is primarily associated with cell proliferation [19,20], our approach was to determine the function of pSTMN1^S16^ on cell cycle progression, proliferation, and metastatic potential, and the impact of the other three serines (S25, S38, and S63) in modulating pSTMN1^S16^-mediated proliferation. Herein, our study shows, for the first time, that both tSTMN1 levels and pSTMN1^S16^ are regulated by HGF/MET during the cell cycle. In addition, pSTMN1^S16^ alone is a primary regulator of tSTMN1 expression, cell proliferation, and cell cycle progression, similar to that observed in response to HGF/MET signaling. These findings were confirmed by substituting S16 with glutamic acid (E) to mimic pSTMN1^S16^ activity. Moreover, pSTMN1^S16^ predominantly regulates proliferation, as it does not initiate or increase metastatic potential in vitro.

## 2. Materials and Methods

### 2.1. Prostate and Breast Cancer Clinicogenomics

Four datasets from cBioPortal (RRID:SCR_014555) and two datasets from TCPA were queried for the clinicogenomic analysis in our study [36,37]. While PCa is a male-specific disease and BC occurs primarily in females, the most common metastatic sites for both cancer types include bone and liver [38]. Therefore, we postulated that *STMN1*, *MET*, and *HGF* would exhibit similar expression profiles in metastatic PCa and BC. For PCa analysis, we selected the Fred Hutchinson Cancer Research Center study on prostate adenocarcinoma [39] with expression microarray (accession number GSE77930; n = 122) and clinical annotations including metastatic organ sites, the TCPA reverse phase protein array dataset of prostate adenocarcinoma samples (n = 425) and breast invasive carcinoma (n = 877), the SU2C-PCF Dream Team study on mCRPC [40] with clinical annotations and gene expression (n = 240), and the Memorial Sloan Kettering study on prostate adenocarcinoma [41] with clinical annotations including metastatic organ sites, castration resistance status, and overall survival. Analyses included gene expression correlation (Spearman’s), gene expression comparison by site using one-way ANOVA, survival analysis using Kaplan–Meier plots with Log-Rank statistics, and gene expression comparison by chemotherapy treatment using Student’s *t*-test. Data were visualized using the R package ‘ggplot2′ v.3.5.1 in R version 4.3.0 [42,43]. For the BC analysis, we used the ROC Plotter tool (ROCplot, RRID:SCR_025347) and the filter selections ‘taxane chemotherapy treatment’ and ‘node positive nodal status’ to query the ROCplot dataset on breast cancer (n = 489) [44] for STMN1, HGF, MET, and STMN1/MET signatures. Of note was the fact that a BC study with data on metastatic organ sites was not found, and therefore it was not possible to query individual metastatic organ sites for BC. Analyses were output as box–whisker plots and compared using Mann–Whitney test and receiver–operator characteristic plots with area-under-curve integration. Additional patient sample information is provided in the Results Section 3.1, Section 3.2 and Section 3.3.

### 2.2. Cells and Reagents

The DU-145 (ATCC # HTB-81, RRID:CVCL_0105) and NMuMG (ATCC # CRL-1636, RRID:CVCL_0075) cell lines were obtained from American Type Culture Collection. HGF (Cat. # 100-39H) was purchased from PeproTech. AMG-337 (Cat. # HY-18696) was purchased from MedChemExpress (Princeton, NJ, USA). Lipofectamine™ 2000 (Cat. # 11668019) was purchased from Thermo Fisher Scientific (Invitrogen, Carlsbad, CA, USA). Polycarbonate track-etch (PCTE) membranes (pore size 8 µm, AP48) for the Neuro Probe chemotaxis chamber were purchased from Neuro Probe. Antibodies against STMN1, STMN1^pS16^, Cyclin A2, Cyclin B1, Cyclin D1, Cyclin E1, GAPDH, HGF, Met, pMET, and p21 are provided in Table S1. The pECFP-N1. STMN1 (a.k.a. stathmin-CFP; Addgene plasmid #86783; RRID:Addgene_86783) was purchased from Addgene (Watertown, MA, USA). The pECFP-N1 empty vector was generated by deleting the *STMN1* gene and the sequence was verified. The MISSION^®^ pLKO.1-puro Non-Target shRNA control (Millipore Sigma Burlington, MA, USA; Cat # SHC016) and pLKO/shSTMN1 (Millipore Sigma, shRNA Clones, STMN1, TRC Clone ID: TRCN0000281292) plasmids were purchased from Millipore Sigma and characterized previously [45]. The STMN1 substitution mutations S16A, S16E, S25A, S25E, S38A, S38E, S63A, S63E, S (16,38) E, S (16)A/(25,38,63) E, S (16)E/(25,38,63) A, S (16,25,38,63) A, and S (16,25,38,63) E were generated using the Quikchange Lightning Site Directed Mutagenesis Kit (Agilent, Santa Clara, CA, USA; Part #210519), as per the manufacturer’s protocol. A diagram outlining the positions of the four serines and the nomenclature for all of the substitution mutations generated in this study is provided in Figure S4C, and the primer sequences for generating these substitution mutations are provided in Table S2.

### 2.3. qPCR

Forward and reverse primers for generating the *STMN1* substitution mutations (Table S2) were designed using the Agilent QuikChange Primer Design program (RRID:SCR_025323). All plasmids were sequence-verified by the DNA Sequencing and Genotyping Core, Cincinnati Children’s Hospital Medical Center.

### 2.4. Cell Viability Assays

Cell growth assays were performed as published previously [45,46]. Briefly, cells were plated at 2 × 10^4^ cells/mL/well in 24-well plates in complete medium (Minimum Essential Medium with Earle′s Balanced Salts (Millipore Sigma, Cat. # 56419C), 10% fetal bovine serum (Cytiva HyClone™, Wilmington, DE, USA; Cat. # SH30071.03), 1% penicillin/streptomycin (Thermo Fisher Scientific, Cat. #: 15140122) overnight, followed by replacement of medium (serum free or 1% FBS as indicated) and incubation for 12–18 h. Cells were treated with fresh medium containing vehicle control or 25 ng/mL HGF −/+ 25, 100, 500 ng/mL AMG-337 for 72 h and viable, and non-viable cell numbers were determined (Trypan Blue Exclusion Test of Cell Viability, Thermo Fisher Scientific, Cat. # 15250061).

### 2.5. Transfection Assays

Transfection assays were performed as published previously [45,46]. DU-145 and NMuMG cells were transfected with a control plasmid (pECFP-N1 empty vector control or pLKO.1-puro Non-Target shRNA control), pECFP-N1.STMN1 (wt human STMN1), pLKO.1/shSTMN1, or pECFP-N1.STMN1 containing a *STMN1* serine substitution mutation using a standard Lipofectamine 2000 protocol (Invitrogen, Carlsbad, CA, USA). Cells were treated with vehicle control or 25 ng/mL HGF −/+ 100 ng/mL of the MET inhibitor AMG-337 as indicated and harvested for analysis.

### 2.6. Doubling Time Assays

DU-145 and NMuMG cells were transfected prior to synchronization, then synchronized using a double thymidine block to obtain >75% in early S phase [47,48] and plated at 7.5 × 10^4^ cells/well in 24-well plates. The following day, cells were treated as indicated, harvested each day for 6 consecutive days, and viable and non-viable cells were counted (Trypan Blue Exclusion Test). The protocols for establishing a double thymidine block and cell treatment are outlined in Figure S4.

### 2.7. Migration and Invasion Assays

The Neuroprobe migration and invasion assays, using non-coated and Corning^®^ Matrigel^®^ Growth Factor Reduced (GFR) (Life Sciences, Glendale, AZ, USA; Cat. #354230)-coated membranes, respectively, were performed as described previously [45].

### 2.8. Western Blot Analysis

Cells (asynchronous or synchronized as indicated) were harvested using RIPA buffer (Invitrogen Inc., Cat. # R0278;) with 1% protease inhibitor cocktail (Thermo Fisher Scientific, Cat. # NC0939492;) and phosphatase inhibitor cocktail (Thermo Fisher Scientific, Cat. # 53-913-110VL;), cellular debris was removed, and protein concentrations were quantified (BCA protein assay, Abcam, Cambridge, UK; Cat. # 102536). Proteins were separated by 10% SDS-PAGE (40 or 50 µg protein/lane), transferred to polyvinylidene difluoride membrane, and blocked for 1 h at room temperature in 3% non-fat dry milk in Tris buffered saline/0.1% Tween. Membranes were probed with primary antibody at 4 °C overnight. A peroxidase conjugated secondary antibody was added at a 1:10,000 dilution for both rat and mouse antibodies, and blots were developed using an Enhanced Chemiluminescence kit (Pierce/Thermo Fisher Scientific, Cat # 32132). For each experiment, the membranes were placed into the same container and probed in sequence, first with phospho-antibodies followed with antibodies to total protein, and lastly with GAPDH (as indicated in the figure legends). Antibodies and dilutions are listed in Table S1. Nocodazole (Thermo Fisher Scientific, Cat # AC358240100) was selected as a positive control for STMN1S16 phosphorylation, since companies use Nocodazole to validate their anti-pSTMN1S16 antibodies (e.g., Thermo Fisher Scientific, Cat. # PA5-17091, RRID:AB 10979092) on Western blots.

### 2.9. Flow Cytometry

Cells were harvested, washed 1x in cold PBS, and fixed with 1:2 cold PBS:Ethanol for at least 2 h at 4 °C. PBS:Ethanol was removed by centrifugation, and cells were resuspended in 1 mL cold PBS followed by the addition of 1 µL RNase A (Thermo Fisher Scientific, Cat. # EN0531), a 30 min incubation for 30 min at 37 °C, and the addition of 100 µL Propidium Iodide (PI; Thermo Fisher Scientific, Cat. # P1304MP) to stain cell nuclei. Stained cells were sorted using the Luminex Guava^®^ Flow Cytometer (Cytek Biosciences, Austin, TX, USA), and sample readouts were analyzed using FlowJo v.10 software, RRID:SCR_008520, to create cell cycle distribution histograms for analysis.

### 2.10. Quantification and Statistical Analysis

Statistical analysis and quantification of significant differences between experiments with multiple treatments were analyzed using one-way ANOVA, applying Dunnett’s post hoc correction for multiple comparisons in GraphPad Prism version 9.4.0 (RRID:SCR_002798). Number of culture plates or wells/treatment, n = 4. Number of repeats per assay, n ≥ 3. Significance was determined at *p* < 0.05.

## 3. Results

### 3.1. Increased STMN1 Is Associated with Increased MET and HGF in mCRPC

**Figure 1:**
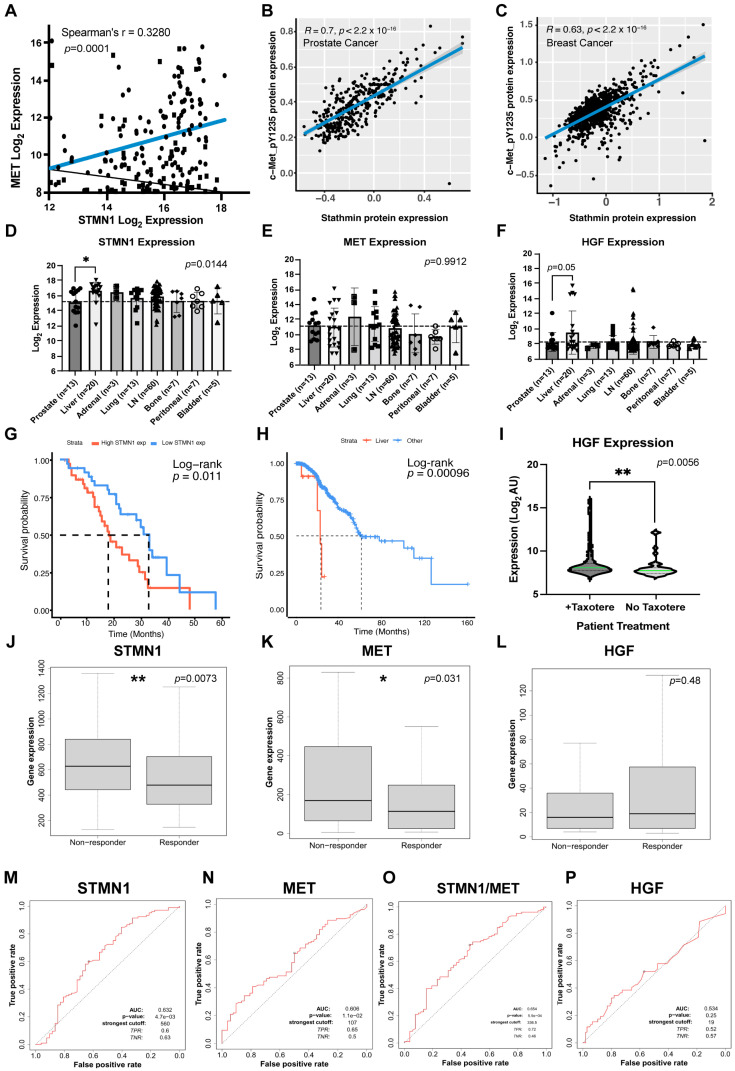
STMN1 and HGF/MET expression co-varies in clinical samples of mCRPC and mBC. (**A**) Correlation analysis of MET and STMN1 gene expression in mCRPC [accessed via cBioPortal, expression microarray and limited clinical annotations, accession number GSE77930 3] (n = 133); (**B**) Correlation analysis of cMETpY1235 and STMN1 protein expression in mCRPC (TCGA-PRAD cohort 48 (n = 425); (**C**) Correlation analysis of cMETpY1235 and STMN1 protein expression in mBC (TCGA-BRCA cohort 49 (n = 877); (**D**) STMN1, (**E**) MET, and (**F**) HGF expression in PCa samples taken from primary or mCRPC sites (GSE77930 cohort 3); (**G**) Kaplan–Meier plot of patients with castration resistant PCa (CRPC) overall survival stratified by gene expression of STMN1 from the SU2C/PCF Dream Cohort (High STMN1 exp; n= 40, Low STMN1 exp; n = 41) [accessed via cBioPortal, whole exome sequencing and clinical annotations]; (**H**) Survival of patients with CRPC with liver metastases (Liver; n = 6) or not (Other; n = 234) [accessed via cBioPortal, mutation/alteration data with clinical annotations but no expression data]. Curve comparisons made using Log-Rank statistics; horizontal line indicates 0.50 probability, and vertical line indicates where each curve reaches 0.50 probability (which is the median value, each listed on graph near respective curves); (**I**) HGF expression when stratifying samples in this dataset based on whether the patient received chemotherapy or not prior to sample acquisition. HGF expression increased significantly following Chemotherapy; (**J**) STMN1, (**K**) MET, and (**L**) HGF expression in tumors of mBC patients receiving taxane treatment stratified by response (Responder; n = 110) or lack thereof (Non-responder; n = 379) [accessed via ROCplot, expression microarray from a single specimen per patient and clinical annotations including response to chemotherapy]; (**M**), STMN1, (**N**) MET, (**O**) STMN1 and MET combined, and (**P**) HGF expression capacity to predict response to taxane treatment in mBC patients determined through receiver–operator characteristic (ROC; area under curve; AUC) analysis. Statistical significance in the respective statistical tests were depicted as * *p* < 0.05, ** *p* < 0.01.

A microarray dataset from PCa tissues and mCRPC lesions found in liver, adrenal gland, lung, lymph node, bone, peritoneal cavity, and bladder sites acquired by rapid autopsy (171 tumors from 63 men) [39] was analyzed to determine whether or not *STMN1* correlated with *MET* and *HGF* gene expression. All patients received androgen-deprivation therapy, and following disease progression most patients also received at least one additional AR pathway-targeted agent (most commonly bicalutamide and flutamide), and at least one systemic chemotherapy (most commonly DTX) [39]. We determined a positive correlation between *STMN1* and *MET* expression (*p* = 0.0001), implying that *STMN1* and *MET* gene expression were co-regulated (Figure 1A). At the translational level in an independent Prostate Adenocarcinoma cohort (350 tumors) from The Cancer Proteome Atlas (TCPA) consortium [49,50], reverse phase protein array analysis demonstrated that STMN1 protein expression and phosphorylated MET-pY1235 protein expression showed a strong positive correlation (*p* = 2.2 × 10^−16^) (Figure 1B). Similar results were observed in the TCPA Breast Cancer cohort (876 tumors; Figure 1C). Together, these observations imply that the STMN1 and MET proteins are stronger indicators of a positive correlation than their gene expression. In addition, *STMN1*, *MET*, and *HGF* were expressed at similar levels in primary PCa tissues and mCRPC lesions in bone, adrenal gland, lung, lymph node, peritoneum, and bladder (Figure 1D–F), inferring that the levels of *STMN1*, *MET*, and *HGF* expression were independent of any observed tumor heterogeneity between men.

### 3.2. Increased STMN1 and HGF Expression Is Associated with Liver Metastasis and Decreased Overall Survival

When *STMN1*, *MET*, and *HGF* expression in mCRPC lesions in liver were compared to the other metastatic sites, *STMN1* (*p* = 0.001) and *HGF* (*p* = 0.05), but not *MET* expression, were higher in mCRPC lesions in the liver (Figure 1D–F). Whether or not the levels of *STMN1*, *MET*, and *HGF* gene expression are interdependent remains to be established. However, an increase in STMN1 and HGF expression in metastatic liver lesions (Figure 1D–F) could correlate with MET via function, in that increased HGF would likely increase MET phosphorylation which, in turn, would phosphorylate STMN1 to regulate its activity. To further define the association of *STMN1* with PCa mortality, we analyzed a SU2C-PCF Dream Team dataset comprised of gene expression and survival data from a cohort of 81 patients with mCRPC [40] and stratified them based on median *STMN1* gene expression. This analysis revealed that mCRPC patients with high *STMN1* expression had a poorer overall survival (median of 17.71 months, *p* = 0.011) compared to those with low *STMN1* expression (median of 33.02 months; Figure 1G). In a second dataset from the Memorial Sloan Kettering cohort, providing survival parameters for mCRPC patients [41], stratification of a castration resistant subset of 240/424 patients with mCRPC based on the presence of liver metastases showed a poorer median overall survival of 23.23 months (*p* = 0.00096) in patients with liver metastases compared to patients with other types of metastases (median overall survival of 61.37 months; Figure 1H). Together, these observations imply that *STMN1* gene expression promotes mCRPC progression and plays a significant role in patient mortality. Additionally, the liver provides a favorable microenvironment for the establishment of metastatic lesions having high *HGF* and *STMN1* expression levels.

We next examined *STMN1* expression in mCRPC samples from patients treated with/without chemotherapy in the Memorial Sloan Kettering cohort [41], primarily DTX, which targets microtubule dynamics (a cellular function regulated by STMN1). Unexpectedly, *HGF* expression was greater (*p* = 0.001) in patients receiving chemotherapy after androgen deprivation therapy (ADT) and second-line therapy (e.g., bicalutamide or flutamide) as compared to those receiving ADT only (Figure 1I), suggesting that chemotherapy may upregulate *HGF* gene expression.

### 3.3. STMN1 and MET Predict Response to Taxane Chemotherapy in Node-Positive Bc Patients

Hormonally dependent cancers, such as PCa and BC, share similarities in biology, cancer progression, and potentially response to therapy [51]. Since taxanes are commonly used to treat other hormonally dependent cancers, we also analyzed the link between *STMN1*, *HGF*, *MET*, and *STMN1*/*MET* gene expression signatures and taxane treatment response in an mBC patient cohort using the ROCplot dataset on BC [44]. Interestingly, *STMN1* (*p* = 0.0073) and *MET* (*p* = 0.031), but not *HGF*, expression was higher in patients who remained unresponsive to paclitaxel (Figure 1J–L). Moreover, *STMN1* and *MET*, but not *HGF*, expression showed potential capacity to predict taxane response in node-positive BC patients (AUC = 0.632 and AUC = 0.606, respectively), and a combined signature improved this score (AUC = 0.654), whereas *HGF* showed no discernable predictive capacity (Figure 1M–P). While these AUC scores are modest, it is representative of a cohort of 489 mBC patients who are not stratified according to disease subtypes. Thus, these scores may potentially be improved by contextualizing mBC biopsy specimens according to metastatic sites. However, mBC biopsy specimens from different metastatic sites were not available; therefore, a comparison of *HGF* gene expression following chemotherapy or *STMN1*, *MET*, and *HGF* expression levels between different metastatic sites could not be done.

Together, the mCRPC and mBC studies suggest that, when *STMN1* and *MET* expression are high, response to taxane chemotherapy is poor, and while responsiveness to taxane is similar, the effectors promoting this response may be selective, as seen by *STMN1* and *HGF* expression being higher in mCRPC, while *STMN1* and *MET* are higher in mBC. The mechanisms underlying the difference in HGF and MET expression are unknown. HGF is abundantly expressed in the microenvironment of metastatic prostate cancer in the bone, and high HGF levels are often associated with metastatic PCa to bone, suggesting that HGF is a key component of regulating HGF/MET signaling in mCRPC [30]. While HGF also plays a significant role in mBC, MET can heterodimerize with the epidermal growth factor receptor (EGFR) and c-Src, leading to c-Met phosphorylation in the absence of HGF [52]. Thus, in mBC, factors that activate EGFR would also phosphorylate MET, making MET less dependent on HGF for activation and signaling. Moreover, subdividing patients with mCRPC (which highly express *STMN1*) further into “high” and “low” STMN1 subgroups could serve as a prognostic indicator of overall survival in these groups of patients. Since the co-regulation of STMN1 with HGF and MET protein expression is indicative of a coordinated mechanism, the differential phosphorylation of STMN1 was evaluated to determine its impact on cancer progression and metastasis.

### 3.4. STMN1^S16^ Phosphorylation and Cell Proliferation Are Regulated by HGF/MET

**Figure 2:**
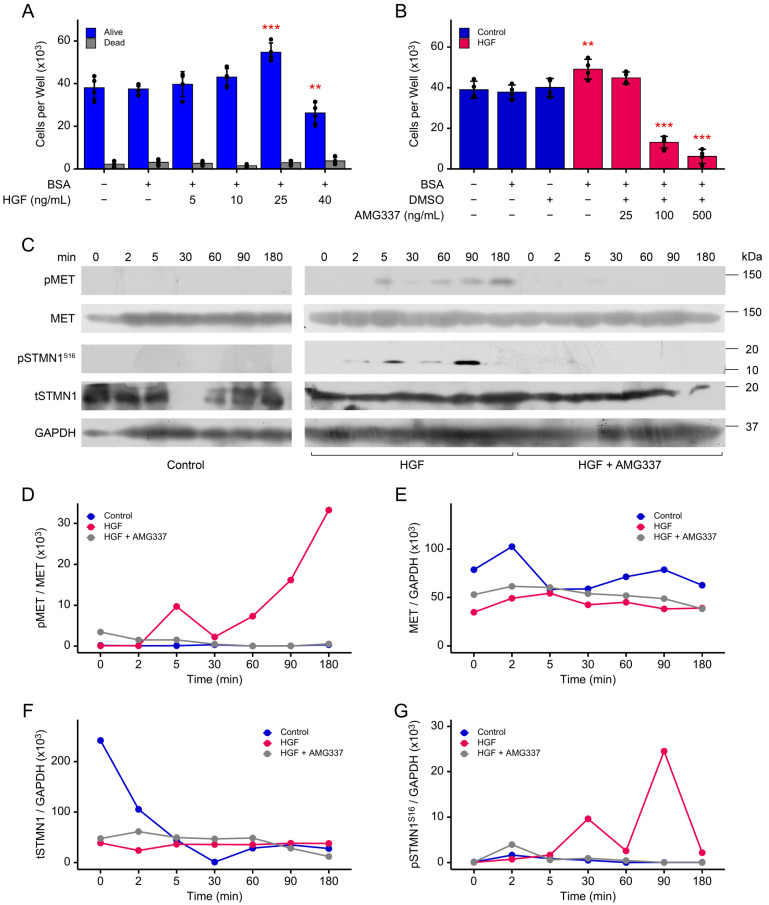
HGF treatment promotes MET and STMN1S16 phosphorylation and cell growth. (**A**) Asynchronous DU-145 cells were treated with increasing HGF concentrations as indicated for 72 h. Live and dead cell numbers were determined using the Trypan Blue Exclusion Test of cell viability; (**B**) Asynchronous DU-145 cells were treated with 25 ng/mL HGF ± 25, 100, or 500 ng/mL AMG337 for 72 h, and cell number was determined using the Trypan Blue Exclusion Test. Treatment replicates, n = 4; independent experimental replicates, n = 3; and data are presented as the mean ± SD. Statistical comparisons were made using one-way ANOVA, applying Dunnett’s post hoc correction for multiple comparisons where ** *p* < 0.01, *** *p* < 0.001; (**C**) The Western blots represent one complete experiment, where asynchronous DU-145 cells were treated with vehicle control, 25 ng/mL HGF, or 25 ng/mL + 100 mg/mL AMG337 for the times indicated; the blots/membranes were placed into the same container and probed in sequence first with anti-pSTMN1 Ser16, anti-pMET, anti- anti-STMN1, MET, and finally anti-GAPDH antibodies. The blots for pMET (**D**), MET (**E**), tSTMN1 (**F**), and pSTMN1S16 (**G**) were analyzed by densitometry (ImageJ software: https://imagej.net/ij/, accessed on 9 July 2025). Given that tSTMN1 levels varied down to undetectable levels (densitometry value = 0) in response to HGF/MET activity, it was not possible to compare pSTMN1^S16^/tSTMN1. Therefore, pSTMN1^S16^ levels were compared to the GAPDH control as this analysis most closely represented the intensity of pSTMN1S16 expression on the Western blots. Independent experimental replicates, n = 3. The original Western Blots can be found in Supplementary File S1.

STMN1^S16^ phosphorylation is associated with cancer cell proliferation [20], and HGF is a stromal cell growth factor that promotes DU-145 PCa cell proliferation [26]. To further confirm the stromal cell expression of HGF, using 10 paired prostate cancer tissues with adjacent normal prostate tissue samples, we analyzed a single cell transcriptomic dataset of prostate cancer samples [53]. Tuong et al. identified 14 cell clusters containing cells from both normal and cancer samples. These included stromal cells (endothelial and fibroblasts) and immune cells, as well as several subtypes of epithelial cells (luminal epithelial, LE; basal epithelial, BE; club epithelial, CE; hillock epithelial, HE). Of these epithelial cell clusters, two subclusters of LE cells (LE KLK3+ and LE KLK4+) were representative of prostate tumors. HGF gene expression was only observed in fibroblasts, mononuclear phagocytes, and endothelial cells (Figure S1A). Moreover, we confirmed that the DU-145 cells themselves do not express HGF, using the Cancer Cell Line Encylopedia transcriptomic dataset (Figure S1B). After determining that HGF is exogenously supplied to prostate cancer cells, the following experiments were performed to determine the impact of HGF/MET activity on the proliferation and phosphorylation of MET and STMN1. In asynchronized DU-145 cells, HGF promoted DU-145 cell proliferation similar to that reported by Humphrey et al. [26], and growth was optimal at 25 ng/mL HGF (*p* < 0.01) (Figure 2A). In addition, cells maintained a classic cobblestone-like morphology (Figure S2 and [45]). In contrast, proliferation decreased at 40 ng/mL HGF (*p* < 0.001); however, cells remained viable and exhibited a spindle shape-like morphology reminiscent of the scatter factor properties of HGF [26]. Therefore, 25 ng/mL HGF was used in subsequent experiments to ensure that cells exhibited typical DU-145 proliferation and contact inhibition characteristics. In addition, cell growth decreased (*p* < 0.001) when cells were treated with HGF and 100 ng/mL AMG337 [54], a highly selective small-molecule inhibitor of MET in advanced solid tumors (Figure 2B).

Analysis of HGF-mediated MET phosphorylation (pMET) determined that pMET was induced at 5 min post HGF treatment and peaked at 180 min (~132- and 180-fold increase over control, respectively), and AMG337 inhibited MET phosphorylation to nearly undetectable levels (Figure 2C,D). Similarly, HGF induced pSTMN1^S16^ at 5 min (~398-fold increase over control), with peaks at 30 min (~815-fold increase over control) and 90 min (~1107-fold increase over control), and as above, AMG337 inhibited pSTMN1^S16^ to nearly undetectable levels (Figure 2C,G). In contrast, HGF did not regulate tMET (Figure 2E) or tSTMN1 (Figure 2F) levels; however, in the control groups, tMET levels appeared modestly biphasic, while tSTMN1 levels decreased over 30 min and did not return to basal control levels by 180 min. Together, these observations imply that, in asynchronous cells, basal tMET and tSTMN1 levels were modulated over time, and that HGF primarily regulated cell proliferation and pMET and pSTMN1^S16^ in a time-dependent manner.

Early studies report that STMN1 is phosphorylated at S16 by the Ca^2+^/calmodulin-dependent protein kinase II (CaMKII), and that this regulates spindle and microtubule formation in Jurkat T, HeLa, HepG2, and MCF7 cells [9,23,55]. While CaMKII is most frequently cited as regulating STMN1^S16^ phosphorylation, this does not occur in all cell types, as STMN1^S16^ is not phosphorylated by CAMKII in K562 lymphoblast cells [56]. Therefore, to determine whether or not HGF/MET activity was a primary mechanism for phosphorylating STMN1^S16^ in DU-145 cells, or whether or not CaMKII could also phosphorylate STMN1^S16^, DU-145 cells were treated with the CaMKII activator, oleic acid. Nocodazole, an established inducer of STMN^S16^ phosphorylation, was also used as a positive control, (a standard established by companies such as Thermo Fisher, Cell Signaling Technology, and Abeomics, to validate their phosphorylated STMN^S16^ antibodies). Oleic acid had no effect on phosphorylating STMN1^S16^, while nocodazole increased pSTMN1^S16^ in a dose dependent manner, indicating that CAMKII activity did not phosphorylate pSTMN1^S16^ (Figure S3). Together, these findings imply that CaMKII-mediated STMN1 phosphorylation is cell/tissue-specific.

### 3.5. Both pSTMN1^S16^ and tSTMN1 Levels Are Modulated by HGF/MET During Cell Cycle Progression

**Figure 3:**
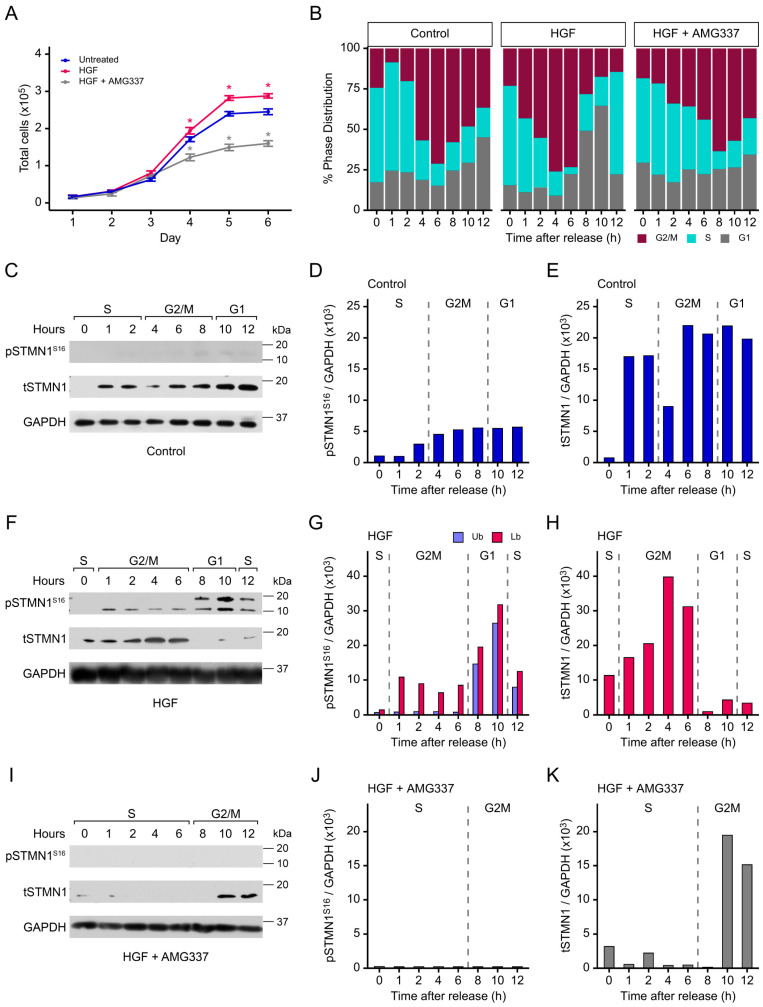
HGF/MET activity regulates cell doubling and modulates pSTMN1^S16^ and tSTMN1 levels during cell cycle progression. DU-145 cells were synchronized in the early S phase using a double thymidine block (as outlined in Figure S4A) and treated with vehicle control, 25 ng/mL HGF, or 25 ng/mL HGF + 100 mg/mL AMG337 for the times indicated; (**A**) Cell doubling time. Treatment replicates, n = 4; independent experimental replicates, n = 3; data are presented as mean ± SD and statistical comparisons were made using one-way ANOVA applying Dunnett’s post hoc correction for multiple comparisons, where * *p* < 0.0001; (**B**) Flow cytometry was performed and % cell populations in S, G2/M, and G1 were determined (FlowJo software Flow Cytometry gating strategy can be found in Figure S5. The times spent in the G1, S, or G2 phases are marked above the Western blots to determine where in the cell cycle pSTMN1^S16^ and tSTMN1 were expressed. The Western blots represent one complete experiment, where cells were treated with vehicle control (**C**), HGF (**F**), and HGF+AMG337 (**I**); the blots/membranes were placed into the same container and probed first with the pSTMN1 Ser16 antibody, followed by anti-STMN1 and then anti-GAPDH antibodies. The HGF-treated group clearly expressed both pSTMN1^S16^ and tSTMN1, and these blots served as positive controls for the vehicle control and HGF+AMG337 blots expressing low/no levels of pSTMN1^S16^ or tSTMN1. The relative levels of pSTMN1^S16^ and tSTMN1 expression in vehicle control (**D**,**E**), HGF (**G**,**H**) and HGF+AMG337 (**J**,**K**) were determined by densitometry (ImageJ software). Given that total STMN1 expression levels varied down to undetectable levels (densitometry value = 0) in response to HGF/MET activity during the cell cycle, it was not possible to compare pSTMN1^S16^/tSTMN1. Therefore, pSTMN1^S16^ levels were compared to the GAPDH control as this most closely represented the band intensity of pSTMN1^S16^ expression on the Western blots. Independent experimental replicates, n = 3. The original Western Blots can be found in Supplementary File S1.

Since STMN1^S16^ phosphorylation is primarily associated with cell proliferation [19,20], we postulated that HGF/MET regulated STMN1^S16^ phosphorylation in a time/ cell cycle phase dependent manner. Moreover, little is known of the regulation of tSTMN1 expression during the cycle. Therefore, the following experiments were performed to determine the interrelationship between tSTMN1 levels, STMN1^S16^ phosphorylation, and HGF/MET-mediated signaling during proliferation and cell cycle progression. To determine the impact of HGF/MET on cell doubling time, DU-145 cells were synchronized in the early S phase using a double thymidine block, as outlined in Figure S4 [47,48,57]. Treatment with HGF shortened cell doubling time to 22.7 h (*p* < 0.01), while the addition of HGF+AMG337 lengthened doubling time to 32.4 h (*p* < 0.01) as compared to the vehicle control group (25.2 h) (Figure 3A). When cells were treated with HGF, flow cytometric analysis showed that the % phase distribution of cells in the second S phase at 12 h was similar to that in the first S phase at 0 h when cells were released from the double thymidine block, indicating that HGF-treated cells had completed one cell cycle within 12 h (Figure 3B). Furthermore, analysis of the individual time points revealed that HGF shortened the cell cycle by 4 h compared to the vehicle control group, with cells entering G2/M at 1 h, exiting into G1 at 8 h, and entering the second S phase at 12 h. In contrast, HGF+AMG227 lengthened the cell cycle by extending the S phase to 6 h, with cells entering into G2/M at 8 h and remaining arrested in G2/M at 12 h. In addition, the % phase distribution of cells in G1 remained relatively constant during this time period.

pSTMN1^S16^ and tSTMN1 protein levels were also determined during cell cycle progression. In the vehicle control group, pSTMN1^S16^ was detected at low levels during G2/M and G1 (Figure 3C,D). However, after treatment with HGF, pSTMN1^S16^ was detected at 1 h as cells entered G2/M and remained phosphorylated throughout G2/M, as expected for mitosis [58], and then increased as cells exited into G1 at 8 h (Figure 3F,G). In addition, a higher molecular weight band appeared during G1 and decreased as cells entered into the second S phase. At least twelve increasingly phosphorylated STMN1 isoforms have been identified; therefore, the higher bands likely contain other phosphorylated serines (i.e., S25, S38, and/or S63) in addition to pSTMN1^S16^ [11,56,59]. In contrast, the addition of AMG337 inhibited the HGF-induced pSTMN1^S16^ (Figure 3I,J).

In contrast, in the control group, tSTMN1 was initially detected in the S phase at 1 h following release from the thymidine block, with a modest decrease at 4 h when entering G2/M before increasing at 6 h to a constant level through G1 (Figure 3C,E). With HGF treatment, tSTMN1 was already detected in the S phase at 0 h, and levels increased in G2/M, followed by a decrease most likely due to the increase in pSTMN1^S16^ isoforms observed in G1 (Figure 3F,H). The addition of AMG337 inhibited tSTMN1 expression to nearly undetectable levels throughout the extended S phase and into early G2/M at 8 h, followed by a rise at 10–12 h (Figure 3I,K). Interestingly, tSTMN1 expression was low/absent at 0 h in the S phase in the control group and during the extended S phase of the HGF+AMG337 group, suggesting that inhibition of tSTMN1 was required to maintain extended S phase.

Collectively, these observations imply that HGF/MET promotes pSTMN1^S16^ in G2/M, the phase in which STMN1 phosphorylation is required to promote mitosis [58], and that additional serines are phosphorylated to exit into G1. While tSTMN1 overexpression is reported to prevent mitotic spindle formation in early mitosis [12], the role of over phosphorylation by HGF treatment remains an avenue to be explored. A possible role of HGF-induced over phosphorylation of STMN1^S16^ may be to disrupt mitosis and facilitate the rapid re-entry of cells into the second S phase. In addition, tSTMN1 is upregulated with HGF treatment. Whether the modulation in tSTMN1 levels is due to HGF/MET-regulated transcription or changes in protein stability/degradation remain to be established.

### 3.6. Cyclins and p21 Are Concomitantly Regulated by pSTMN1^S16^

**Figure 4:**
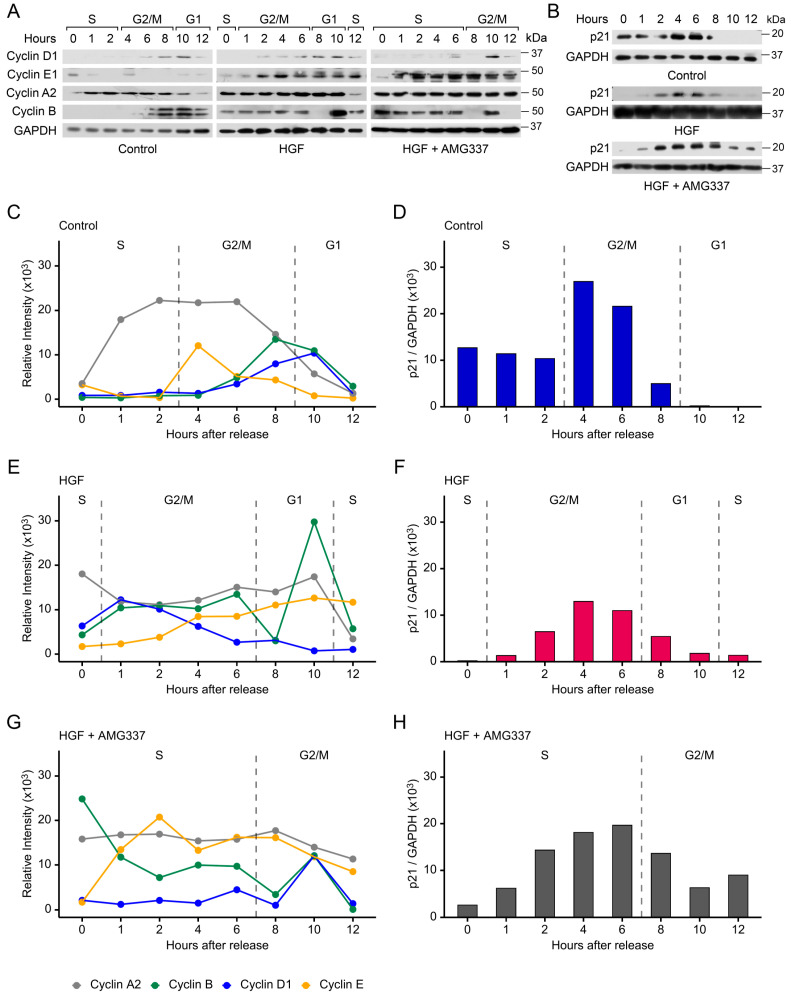
Cyclin and p21 levels are co-regulated with pSTMN1^S16^ during the cell cycle. To dissect the involvement of pSTMN1^S16^ in the cell cycle, DU-145 cells were synchronized in the early S phase using a double thymidine block and treated with vehicle control, 25 ng/mL HGF, or 25 ng/mL HGF + 100 mg/mL AMG337 for the times indicated. The Western blots represent one complete experiment, as described in Figure 3, and Westerns blots were probed with anti-cyclin D1, -cyclin E1, -cyclin A2, -cyclin B1, and -cyclin GAPDH (**A**) and -p21 (**B**) as indicated. Relative intensity of expression was analyzed by densitometry (ImageJ software) in response to vehicle (**C**,**D**), HGF (**E**,**F**), and HGF+AMG337 (**G**,**H**). The approximate times spent in G1, S, or G2/M phases are marked above the Western blots and densitometric analysis. Independent experimental replicates, n = 3. The original Western Blots can be found in Supplementary File S1.

Since growth factors regulate cyclins to facilitate progression into the S phase [25], and HGF promotes cell proliferation and regulates STMN1^S16^ phosphorylation, synchronized DU-145 cells were treated with vehicle control or HGF ± AMG337, and cyclin protein levels were analyzed. In the control group, expression of the cyclins was typical of that expected during cell cycle progression (Figure 4A,C). Cyclin E1, a regulator of entry into the S phase, increased modestly until 4 h and then decreased gradually in G2/M through G1. Cyclin A2, a regulator of the S phase and mitotic entry, increased during S into G2/M and decreased late in G2/M into G1. Cyclin B1, a regulatory protein involved in mitosis, increased to peak later in G2/M at 8 h, and Cyclin D1, which regulates transition from G1 to the S phase, increased in the latter half of G2/M through into G1, but did not transition into a second phase in the absence of growth factor activity.

In contrast, HGF promoted cell cycle progression, resulting in a time-dependent rise in cyclin E1 levels through the S phase, and in parallel, a decrease in cyclin D1 levels from entry into G/2M through to re-entry into the second S phase (Figure 4A,E). Cyclin A2 and cyclin B1 levels remained constant through G/2M, with cyclin A2 decreasing in the second S phase. An exception was the downregulation of cyclin B1 expression to a low level at 8 h, followed by a dramatic peak in expression in G1 at 10 h. These observations suggest that high cyclin B1 levels in combination with low cyclin D1 expression in the G phase could drive the rapid progression of cells into the second S phase. Furthermore, the addition of the AMG337 inhibitor blocked cell cycle progression in G2/M, with cyclin E1 expression rising in the S phase and remaining fairly high in G/2M, while cyclin A2 and cyclin D1 levels remained relatively unchanged in the extended S phase, with cyclin D1 showing a modest peak at 10 h (Figure 4A,G). Importantly, cyclin B1 decreased in the S phase and only showed a modest peak at 10 h compared to that seen in HGF-treated cells.

We also analyzed p21, a cell cycle inhibitor that mediates cell cycle arrest in G1 [60]. In the vehicle control-treated DU-145 cells, p21 expression was expressed in the S phase following release of the double thymidine block and increased during G2/M (4–6 h), followed by a decrease to undetectable levels in G1 (Figure 4B,D). The addition of HGF decreased p21 expression to below detectable levels in the S phase, with p21 levels remaining lower during G2/M compared to vehicle control and decreasing again in G1 and the second S phase (Figure 4B,F). The addition of HGF+AMG337 caused p21 expression to increase throughout the extended S phase and decreased in G2/M (Figure 4B,H). Together, these observations imply that HGF downregulates overall p21 expression to shorten the cell cycle, while the addition of AMG337 increased p21 expression to block DU-145 cells in G2/M.

Whether pSTMN1^S16^ modulates cyclin and p21 expression directly or indirectly remains to be established. However, total STMN1 knockdown resulted in a redistribution of the cell cycle with a decrease in J7 hepatocellular carcinoma cells in the G1 population and a 2-fold increase of J2 cells in the G2/M population [61]. In addition, STMN1 knockdown increased cyclin B, but decreased cyclin D, protein levels. However, the molecular mechanism of these activities remains to be determined [61]. In U251 glioma cells, STMN1 knockdown inhibited both cyclin D1 and cyclin B expression while upregulating p21 expression, leading to cell cycle arrest in vitro [62]. These observations imply that STMN1 directly modulates cyclin D1, cyclin B, and p21 expression, and this is cell-type specific. Our study shows that the patterns of cyclin and p21 expression are similar in cells expressing the pSTMN1^S16E^ substitution mutation as compared to the phosphorylation of STMN1^S16^ in cells treated with HGF, suggesting that STMN1 phosphorylation on S16 may directly modulate cyclin and p21 expression. Further studies are needed to determine the mechanisms by which phosphorylation of the STMN1 serines, alone or in combination, regulates cyclin and p21 expression.

### 3.7. pStathminS16 Alone Is Sufficient for Regulating Cell Cycle Progression and Growth

**Figure 5:**
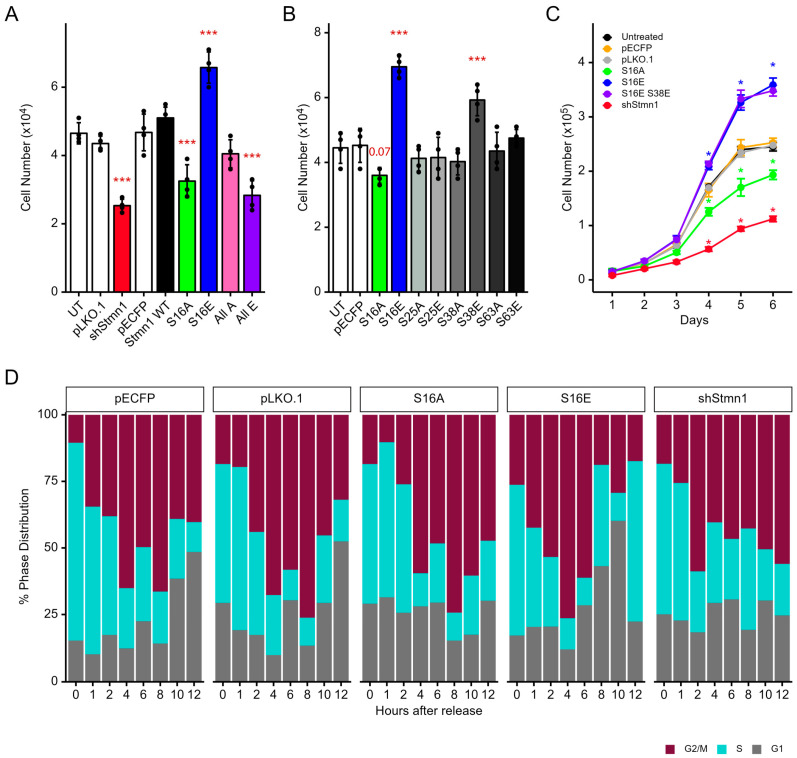
STMN1^S16E^ alone is sufficient for promoting cell cycle progression and growth. (**A**,**B**) Cell proliferation assays. DU-145 cells that were transfected with the pECFP empty vector control, or with pECFP expressing wild type (wt) STMN1, or S16, S25, S38, S63 substitution mutants where S was substituted with alanine (A) or glutamic acid (E) to mimic dephosphorylation and phosphorylation, respectively (detailed in Figure S4C and Table S2). In 4A, all four serines were substituted with A, and in 4E, all four serines were substituted with E. Additional controls: UT, untransfected control; pLKO.1 control (containing scrambled RNA), pLKO.1/shSTMN1 [45]; (**C**) Cell doubling time. DU-145 cells were transfected with the plasmids as indicated and synchronized in the early S phase using a double thymidine block (protocol outlined in Figure S3B). Cells were counted on days 1 through 6 after time of release. Flow Cytometry gating strategy can be found in Figure S5. (**A**–**C**) Treatment replicates, n = 4; independent experimental replicates, n = 3; and data are presented as mean ± SD. Statistical comparisons were made using one-way ANOVA applying Dunnett’s post hoc correction for multiple comparisons where * *p*<0.05, *** *p* < 0.001; (**D**), Flow cytometric analysis (FlowJo software). Cells were transfected with control vectors pECFP or pLKO.1, or STMN1^S16E^, STMN1S^16A^ or pLKO.1/shSTMN1, synchronized in G1/S, and the % cell populations in S, G2/M, and G1 were determined. A representative flow cytometric analysis was presented; independent experimental replicates, n = 3.

A series of plasmids expressing S16, S25, S38, and S63 to alanine (A) or glutamic acid (E) substitution mutations to mimic dephosphorylation and phosphorylation, respectively (Figure S4C, Table S2), were generated to investigate the function of S16 phosphorylation alone, or in combination with S25, S38, and/or S63 phosphorylation, in regulating cell proliferation. STMN1^S16E^ mimicking S16 phosphorylation increased cell proliferation (*p* < 0.001), while STMN1^S16A^ mimicking S16 dephosphorylation decreased cell proliferation (*p* < 0.01) as compared to several controls, including a pECFP empty vector control, a pECFP vector expressing wildtype (WT) STMN1, and an UT (untransfected) control group (Figure 5A). Our previous study characterized STMN1 knockdown using shSTMN1 [45]. Since STMN1 phosphorylation inactivates STMN1 function [11,12], we transfected DU-145 cells with the shSTMN1 plasmid to determine whether or not knocking down STMN1 protein inhibited DU-145 cell proliferation. As seen in Figure 5A, shSTMN1 decreased cell proliferation (*p* < 0.001) compared to a pLKO.1 vector control (containing scrambled RNA). In addition, the 4E mutant where all four serines were substituted with E to mimic total phosphorylation also inhibited cell growth (*p* < 0.01), similar to that observed for shSTMN1, implying that 4E acted as a functional knockdown. In contrast, the 4A mutant representing activated STMN1 where all four serines were dephosphorylated did not significantly affect cell proliferation.

We also compared the activities of the other three serines to STMN1^S16E^ (a.k.a., S16E) and determined that only S38E, but not S25A, S25E, S38A, S63A, or S63E, increased cell proliferation (*p* < 0.01; Figure 5B). This finding was expected as cyclin-dependent kinase 1 (CDK1) phosphorylates STMN1^S38^ [9,10]. Taken together, these observations suggested that STMN1^S16E^ regulated cell proliferation and that STMN1^S38E^ could participate in this process. Therefore, STMN1^S16E^ and STMN1^S38E^ expression were analyzed in combination using cell doubling time to determine whether or not both serines were essential for promoting cell proliferation. In synchronized cells, STMN1^S16E^ shortened doubling time by 2.6 h (*p* < 0.01), while STMN1^S (16,38)E^ shortened doubling time by 3.1 h (*p* < 0.01) compared to the pECFP control; however, these doubling times were not statistically different (*p* = 1.0) (Figure 5C). In contrast, STMN1^S16A^ lengthened cell doubling time by 2.6 h (*p* < 0.01) compared to the pECFP control, and pLKO.1/shSTMN1 lengthened doubling time by 5.8 h (*p* < 0.01) compared to the pLKO.1 control.

In addition, flow cytometric analysis using synchronized cells transfected with STMN1^S16E^, STMN1^S16A^, or shSTMN1 was performed and compared to vector controls and untransfected cells treated with vehicle, HGF, or HGF+AMG337 (in Figure 3B) to determine whether STMN1^S16E^ or STMN1^S16A^ activated or inhibited cell cycle progression. Cells expressing STMN1^S16E^ entered G2/M at 1 h, exited into G1 at 8 h, and entered the second S phase at 12 h (Figure 5D), thus shortening the S phase by 4 h, similar to that observed in HGF-treated cells (Figure 3B). In contrast, cells expressing STMN1^S16A^ delayed entry into G2/M by 4 h and the cells were arrested in G2/M at 12 h (Figure 5D), similar to that observed in HGF+AMG322-treated cells (Figure 3B). In shSTMN1-expressing cells, STMN1 knockdown promoted entry into G2/M at 2 h, and cells remained arrested in G2/M at 12 h, similar to that observed in STMN1^S16A^-expressing cells and HGF+AMG322-treated cells.

Collectively, these data imply that STMN1^S16^ phosphorylation is a key regulator of cell cycle progression. In addition, STMN1^S38^ phosphorylation is secondary to STMN1^S16^ phosphorylation since combining STMN1^S38E^ with STMN1^S16E^ does not increase doubling time over that observed with STMN1^S16E^ alone, and phosphorylation of all four serines (4E) act as an STMN1 functional knockdown, similar to that observed with shSTMN1.

### 3.8. STMN1^S16E^ and STMN1^S16A^ Modulate tSTMN1, Cyclins, and p21 Levels

**Figure 6:**
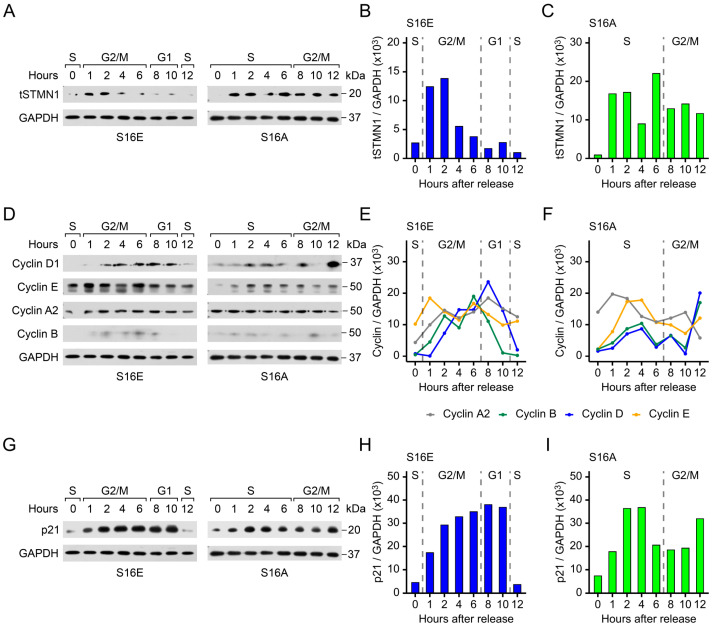
STMN1^S16^ phosphorylation status regulates total STMN1 and cell cycle protein expression. DU-145 cells were transfected with pECFP/STMN1^S16E^ (**B**,**E**,**H**) or pECFP/STMN1^S16A^ (**C**,**F**,**I**), synchronized in the early S phase using a double thymidine block, and collected every h for 12 h after release from the thymidine block. Westerns blots were probed with anti-STMN1 (**A**), anti-cyclin D, -cyclin E1, -cyclin A2, and -cyclin B1 (**D**) and -p21 antibodies (**G**), and the relative intensity of expression was analyzed by densitometry for STMN1 (**B**,**C**), the cyclins (**E**,**F**), and p21 (**H**,**I**). Representative Western blots and densitometry were shown; independent experimental replicates, n = 3. Untransfected cells treated with vehicle, HGF, or HGF+AMG337 in Figure 3 served as controls for tSTMN1 expression to determine whether or not STMN1^S16E^ or STMN1^S16A^ regulated tSTMN1 expression levels. In addition, untransfected cells treated with vehicle, HGF, or HGF+AMG337 in Figure 4 served as controls for cyclin and p21 expression to determine whether or not STMN1^S16E^ or STMN1^S16A^ regulated their expression and activated or inhibited cell cycle progression. The original Western Blots can be found in Supplementary File S1.

To determine whether or not STMN1^S16^ phosphorylation modulated tSTMN1 expression, cells were transfected with STMN1^S16E^ or STMN1^S16A^ and synchronized as outlined in Figure S4B. Untransfected cells treated with vehicle, HGF, or HGF+AMG337 in Figure 3 served as controls for tSTMN1 expression to determine whether or not STMN1^S16E^ or STMN1^S16A^ regulated tSTMN1 expression levels. When cells expressed STMN1^S16E^, tSTMN1 was detected at low levels in the S phase (at 0 h), increased during G2/M, and decreased back down to low levels in G1 and entry into the second S phase (Figure 6A,B), similar to that observed for untransfected HGF-treated cells (Figure 3F,H), albeit at a lower amplitude. When STMN1^S16A^ was expressed, tSTMN1 was detected at 1 h and remained modestly higher up to 6 h, followed by a slight decrease at 8 h onward (Figure 6A,C), in a pattern similar to that in untransfected vehicle control cells (in Figure 3C,E). However, a key difference was that the time STMN1^S16A^ cells spent in the S phase was shifted to the right by 4 h, resulting in G2/M arrest, whereas vehicle control cells exited into G1 at 10 h, demonstrating that dephosphorylation of S16 (as represented by S16A) was sufficient to prevent STMN1^S16A^ cells from completing the cell cycle. A further difference was that, while STMN1^S16A^ cells were arrested in G2/M similar to that observed in untransfected HGF+AMG337 cells (Figure 3I,K), tSTMN1 expression in STMN1^S16A^ cells was considerably higher during the S phase compared to HGF+AMG337 cells, where tSTMN1 levels only increased during G2/M, implying that STMN1^S16A^ deregulated tSTMN1 expression.

In addition, cells were transfected with STMN1^S16E^ or STMN1^S16A^ and synchronized as outlined in Figure S4B, and untransfected cells treated with vehicle, HGF, or HGF+AMG337 in Figure 4 served as controls for cyclin and p21 expression to determine whether or not STMN1^S16E^ or STMN1^S16A^ regulated their expression and activated or inhibited cell cycle progression. A key observation was that the length of the cell cycle for STMN1^S16E^ cells (Figure 5D) and untransfected cells treated with HGF (Figure 3B) were the same, with cells completing the cycle and entering the next S phase at 12 h. In STMN1^S16E^ cells, the pattern of cyclin A2 expression (Figure 6D,E) was similar to that in untransfected cells treated with HGF (Figure 4A,E). Cyclin B1 expression was also similar to that in untransfected cells treated with HGF; and while the peak of expression shifted to 6 h (G2/M) compared to 10 h (G1) in the HGF-treated cells, the length of the STMN1^S16E^ cell cycle remained the same as that of cells treated with HGF. One difference between S16E expression and HGF treatment was that cyclin E1 expression in STMN1^S16E^ cells was high and remained fairly constant throughout the cell cycle, whereas in HGF-treated untransfected cells, cyclin E1 was low in S where it increased steadily through S and G2/M, with the highest levels in G1, as would be expected for the cyclin E1-mediated transition from G1 to the S phase [63]. Another difference was that the regulation of cyclin D1 expression in STMN1^S16E^ cells was more typical of a normal cell cycle [64], with cyclin D1 levels low during the S phase, increasing during G2, remaining high in G1, and then decreasing back down in the second S phase; while in HGF-treated untransfected cells, cyclin D1 decreased in the latter half of G2/M through exit into G1.

Another key observation was that the length of the STMN1^S16A^ cell cycle (Figure 5D) was the same as that of untransfected cells treated with HGF+AMG337 (Figure 3B), where cells in both groups were arrested in G2/M. In STMN1^S16A^ cells (Figure 6D,F), cyclin A2 and cyclin E1 expression was similar to that of untransfected HGF+AMG337-treated cells (Figure 4A,G). Likewise, the pattern of cyclin B1 expression was similar to untransfected HGF+AMG337-treated cells, albeit at a lower amplitude, while cyclin D1 expression appeared elevated compared to untransfected HGF+AMG337-treated cells.

We also analyzed the impact of STMN1^S16^ phosphorylation status on p21 expression. The pattern of p21 expression in STMN1^S16E^ cells during the cell cycle (Figure 6G,H) was similar to that of p21 in HGF-treated cells. In STMN1^S16A^ cells, the pattern of p21 expression during the cell cycle was similar to that observed in HGF+AMG337-treated cells (Figure 6G,I). Of note is that, while the pattern of p21 expression in these groups was similar, the overall amplitude of p21 expression was higher in STMN1^S16E^ and STMN1^S16A^ cells, compared to the HGF and HGF+AMG337 cells, respectively, suggesting that the selective modulation of S16 phosphorylation increased over all p21 levels.

Collectively, the modulation of tSTMN1, the cyclins, and p21 during cell cycle progression, as well as the length of the cell cycle, are sufficiently similar to group S16 phosphorylation (represented by STMN^S16E^) with HGF treatment and S16 dephosphorylation (represented by STMN^S16A^) with HGF+AMG337 treatment. Together, these observations indicate that the S16 phosphorylation signature alone could impact the rate of cell proliferation by regulating tSTMN1, cyclins A2, B1, D1, and E1, and p21 expression.

### 3.9. pSTMN1^S16^ Regulates Cell Proliferation but Not Metastatic Potential

Since HGF and MET play important roles in cancer growth and metastasis [25,27,28], and STMN1^S16^ phosphorylation is regulated by HGF/MET signaling, the NMuMG and DU-145 cell lines were used to determine the impact of STMN1^S16^ phosphorylation on cell growth, migration, and invasion. In contrast to the breast cancer cell lines, the NMuMG mammary gland cell line will undergo epithelial mesenchymal transition (EMT) [65], and they express the MET receptor [66] and low STMN1 levels required to undergo EMT [45]. Similarly, DU-145 cells express the MET receptor, and they undergo EMT when STMN1 levels are low/knocked down [45]. In addition, DU-145 cells only exhibit moderate metastatic potential under basal conditions, allowing for the analysis of factors that promote or inhibit metastatic potential using the same cell line [45,67]. Therefore, these lines were selected to investigate the impact of modulating STMN1^S16^ phosphorylation alone or in combination with other serines on cell growth, migration, and invasion.

**Figure 7:**
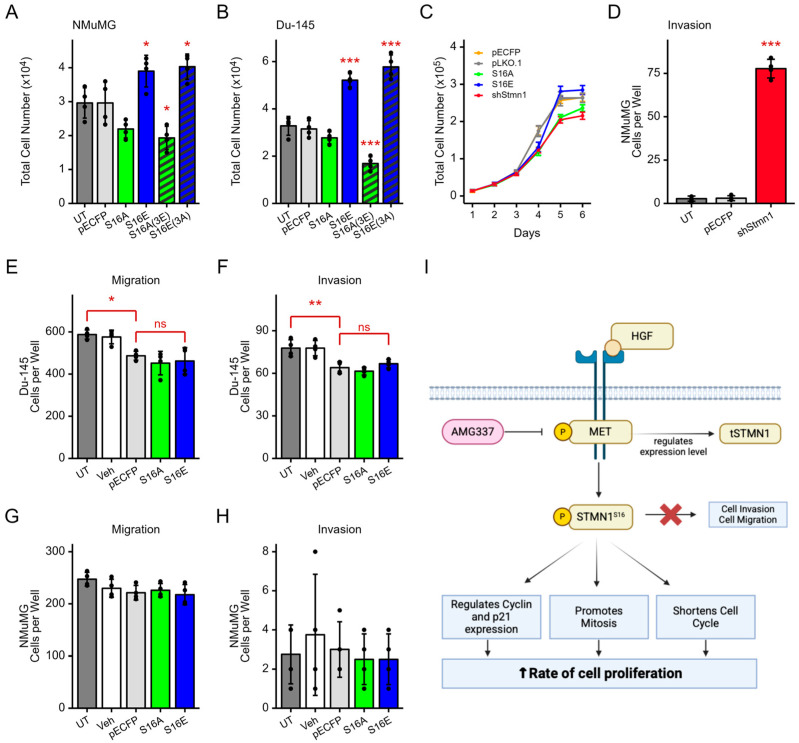
NMuMG and DU-145 cell proliferation, but not metastatic potential, is modulated by STMN1^S16^ phosphorylation. (**A**) NMuMG and (**B**) DU-145 cells were transfected with the indicated plasmids, and cell numbers were determined using the Trypan Blue Exclusion Test; (**C**) NMuMG cell doubling time. NMuMG cells were transfected according to the protocol outlined in Figure S4B and synchronized in the early S phase using a double thymidine block. Cells were counted on days 1 through 6 after time of release; (**D**) NMuMG cells were either treated with vehicle control or transfected with pECFP or shSTMN1 to serve as a positive invasion assay control (**E**,**F**), DU-145 and (**G**,**H**), NMuMG cells were treated with vehicle control, or transfected with pECFP, pECFP/STMN1^S16E^, or pECFP/STMN1^S16A^ as indicated. Neuroprobe migration (**E**,**G**) and invasion (**F**,**H**) assays using non-coated and Corning^®^ Matrigel^®^ Growth Factor Reduced (GFR) (Life Sciences, Cat. #354230)-coated membranes, respectively, were performed as described previously [45]. Images of wells containing migrating or invading cells are provided in Figure S6. Treatment replicates, n = 4; independent experimental replicates, n = 3; data are presented as mean ± SD. Statistical comparisons were made using one-way ANOVA applying Dunnett’s post hoc correction for multiple comparisons for (**A**–**C**) and Tukey’s post hoc correction for multiple comparisons for (**D**–**H**), where ns is not statistically significant, * *p* < 0.05, ** *p* < 0.01, *** *p* < 0.001; (**I**) Diagrammatic representation of the HGF/MET-mediated regulation of STMN1^S16^ phosphorylation in the regulation of cell cycle progression, cell proliferation, and metastatic potential designed using BioRender.com.

NMuMG cells were transfected with plasmids expressing STMN1^S16E^ or STMN1S16E (3A) to determine whether or not dephosphorylation of the other three serines (S25, S38, S63) modulated the effect of S16 phosphorylation (Figure 7A). Cells were also transfected with STMN1^S16A^ or STMN1^S16A (3E)^ to determine whether or not the phosphorylation of the other three serines modulated the effect of S16 dephosphorylation. Controls included untreated cells and cells transfected with empty vectors and cells treated with the vehicle. STMN1^S16E^ and STMN1^S16E (3A)^ expression promoted NMuMG cell proliferation, while STMN1^S16A^ or STMN1^S16A (3E)^ expression inhibited cell proliferation (*p* < 0.01). Similar results were observed when DU-145 cells were transfected with the same plasmids for comparison (*p* < 0.001) (Figure 7B), confirming that S16E promoted proliferation, S16A inhibited proliferation, and activating (3A) or inhibiting (3E) the other serines in combination with S16E or S16A, respectively, did not alter S16-mediated activity. Analysis of doubling time showed that NMuMG cell doubling time decreased by 1.6 h in cells expressing STMN1^S16E^ and increased by 2.4 h in cells expressing STMN1^S16A^ (Figure 7C). While NMuMG doubling time did not reach statistical significance, the trend was similar to that observed in DU-145 cells (Figure 5C).

In addition, DU-145 and NMuMG were transfected with STMN1^S16E^ or STMN1^S16A^ and analyzed in migration and invasion assays to determine whether or not S16 phosphorylation also regulated metastatic potential. Untreated cells, vehicle-treated cells, and cells transfected with an empty vector and treated with the vehicle served as baseline controls, while NMuMG cells transfected with shSTMN1 served as a positive invasion control (Figure 7D) [45]. Given that DU-145 cells exhibited moderate metastatic potential under basal culture conditions [45,67], it was not surprising that DU-145 cells in the control groups migrated readily through the membrane; however, transfection with STMN1^S16E^ or STMN1^S16A^ did not alter the rate of DU-145 cell migration (Figure 7E and Figure S6). Similarly, NMuMG cells showed low basal migration; however, transfection with STMN1^S16E^ or STMN1^S16A^ did not alter basal migration rates (Figure 7G). In addition, DU-145 and NMuMG cells expressing STMN1^S16E^ or STMN1^S16A^ did not show induced invasion through the Matrigel-coated membrane (Figure 7F,H), as compared to the shSTMN1 control, suggesting that STMN1^S16^ did not modulate metastatic potential. Together, these observations imply that pSTMN1^S16^ alone is sufficient to regulate NMuMG and DU-145 cell proliferation, and that pSTMN1^S16^ is not required to regulate metastatic potential. This signaling axis is summarized in Figure 7I.

## 4. Discussion

Our study provides evidence from clinical mCRPC and mBC studies that *STMN1* and *MET* expressions are co-regulated at a transcriptional and translational level, with STMN1 and MET proteins being stronger indicators of a positive correlation than their gene expression, and that response to taxane chemotherapy is poor when *STMN1* and *MET* expression are high. In mCRPC patients who already express high levels of *STMN1*, further dividing them into “high” and “low” subgroups appears to be a more accurate prognostic indicator of overall survival. Further, *STMN1* and *HGF* levels are highest, and overall survival is poorest in mCRPC in the liver compared to bone and other metastatic sites combined.

While bone remains the most common metastatic site for PCa, liver metastasis has gained considerable attention due to its increased incidence [68,69,70]. Liver metastasis appears more resistant to therapy, with a worse median survival of 13.5 months compared to 21.3 months for bone metastases [68]. Our findings substantiate these statistics, wherein patients with mCRPC to the liver had a median overall survival of 23.23 months (*p* = 0.00096) in patients with liver metastases compared to patients with other types of metastases (median overall survival of 61.37 months) (Figure 1H), further emphasizing liver as a more lethal metastatic site. A recent study reported that the pattern of metastatic spread impacted the combinatorial efficacy of enzalutamide and ADT, since this treatment appeared less effective in men with visceral metastases compared to those with bone or lymph node metastases [71]. These clinical observations imply that the metastatic microenvironment plays a broader role in supporting cancer cell survival and metastasis under adverse therapeutic-induced conditions.

Many clinical studies, including those in Figure 1, employ ‘omics approaches such as microarray, RNA seq, and proteomics to determine gene or protein expression. Thus, a positive correlation between *STMN1*, *MET*, and *HGF* expression and metastasis, responsiveness to taxane, and overall survival in prostate and breast cancer patients is based on total *STMN1* expression within these tumors [72,73,74]. However, in addition to primarily epithelial-derived cancer cells, solid tumors contain numerous other cell types that express STMN1, including cancer-associated fibroblasts (CAFs) [75], endothelial, and immune cells [76]. We reported that freshly isolated primary prostate cancer (PCa) cells from patient prostate cancer (PCa) biopsy specimens and the NMuMG cell line expressed low endogenous levels of STMN1, and that these cells exhibited increased metastatic potential in vivo and in vitro, respectively [45]. Moreover, knocking down STMN1 expression in DU-145 cells using an shRNA approach also increased metastatic potential [45]. Collectively, these observations imply that different tumor cell types express varying levels of STMN1, and analysis of STMN1 expression and function at the cellular level would be more reflective of the role STMN1 plays in promoting tumor growth and metastasis.

As most studies typically use tSTMN1 as a predictor of progression, the expression levels of tSTMN1 do not substantively represent the impact of its phosphorylation-mediated biological activity in CRPC and metastasis. For instance, while an increase in tSTMN1 expression is associated with poorer disease-free survival [72,73,74], the phosphorylation signatures of STMN1 may predict therapeutic responsiveness to paclitaxel. STMN1 phosphorylation on S16 or S63 was strongly associated with improved disease-free survival, while phosphorylation on S25 or S38 was associated with poorer disease-free survival in patients with luminal subtype breast cancer [74]. The taxanes paclitaxel and docetaxel stabilized microtubules and induced cell cycle arrest at the G2/M phase [77], and the paclitaxel-induced phosphorylation of S16 stabilized microtubules and induced apoptosis and cell death in the acute lymphoblastic leukemia (ALL) Jurkat and Namalwa cell lines [19]. Our study showed that pSTMN1^S16^ regulated both prostate cancer and mammary gland cell growth but did not increase metastatic potential (summarized in Figure 7H). Moreover, STMN1^S16A^ also arrested cells in G2/M similar to that reported for taxane treatment [77], implying that the S16 phosphorylation signature could predict therapeutic responsiveness. Together, these studies support the concept that selectively modulating STMN1^S16^ phosphorylation could prevent or block taxane resistance or extend the period of responsiveness to taxane-based therapies.

We observed that tSTMN1 expression and STMN1^S16^ phosphorylation were both regulated by HGF/MET signaling, and that the inhibition of MET decreased tSTMN1 expression. Determining the mechanisms by which HGF/MET regulates tSTMN1 gene or protein expression was beyond the scope of this study; however, since MET also regulates gene transcription [78], we would predict that the inhibition of MET would inhibit MET-mediated transcriptional activity, thereby decreasing *STMN1* gene transcription and protein levels and concomitantly increasing the levels of soluble tubulin available for microtubule formation. Only a few regulators of *STMN1* gene transcription have been identified to date. Forkhead box M1 (FoxM1) directly regulates *STMN1* gene transcription to overexpress tSTMN1 and confer resistance to paclitaxel in SKBR3 breast cancer cells [79]. In addition, p53 mutations or loss of p53 expression derepress *STMN1* gene transcription, resulting in tSTMN1 overexpression [80]. Mutagenesis of E2F Transcription Factor 1 (E2F1) binding sites in the *STMN1* gene decreased STMN1 mRNA and protein levels [81]. Another putative mechanism for decreasing tSTMN1 protein levels could be via post-translational modification, e.g., phosphorylation [82]. A study by Gradin et al. reported that STMN1^S16^ phosphorylation was associated with the partial degradation of tSTMN1 protein, and that the down-regulation of tSTMN1 increased polymerized tubulin and microtubule formation in K562 cells [56]. Whether the HGF/MET-mediated regulation of tSTMN1 occurs via transcription or protein degradation remains to be determined.

In the seminal study on HGF function in PCa cells, Humphrey et al. reported that DU-145 cell proliferation increased in response to HGF treatment [26]; however, STMN1 phosphorylation was not evaluated. Indeed, most studies on the HGF/MET-mediated phosphorylation of STMN1 have not used prostate cancer cell lines, nor have they investigated in detail the regulation of both tSTMN1 and STMN1 phosphorylation during cell cycle progression. One study reported that, in Jurkat T, HeLa, and K562 cells, the dual phosphorylation of STMN1^S25^ and STMN1^S38^ was a prerequisite for the phosphorylation of STMN1^S16^ and STMN1^S63^ during mitosis [83]. In endothelial cells, HGF restored the endothelial cell barrier by inducing peripheral microtubule elongation via Rac1/PAK1 activity, resulting in STMN1^S38^ phosphorylation, microtubule assembly, and recovery of the peripheral actin cytoskeleton [84]. In primary human keratinocytes, HGF increased phosphorylated STMN1^S38^ to promote keratinocyte cell proliferation and cell migration through PI3K/AKT and MAPK signaling [85]. In MDA-MB-231 human breast cancer cells, HGF stimulated the transportation of the PAK1/WAVE2 complex to the leading edge of lamellipodia through phosphorylation of STMN1^S38^ [18]. Together, these studies showed that multiple pathways regulate STMN1^S38^ phosphorylation and that this was dependent on cell type. The pathways regulating STMN1 phosphorylation in CRPC remain to be determined.

One reported pathway specifically regulating STMN1 phosphorylation at S16 is Ca^2+^/calmodulin-dependent protein kinase II (CaMKII); and CaMKII phosphorylated STMN1^S16^ regulates spindle and microtubule formation in Jurkat T, HeLa, HepG2, and MCF7 cells [9,23,55]. However, CAMKII did not phosphorylate STMN1^S16^ in K562 cells [56], and likewise, our study showed that CAMKII did not phosphorylate STMN1^S16^ in DU-145 cells. We also determined that STMN1^S16^ phosphorylation did not promote metastasis, unlike that reported for metastatic lung (A549) and head and neck small cell carcinoma (212LN) cells [86]. However, the role of STMN1 phosphorylation and the serines that directly promote metastasis remains surprisingly correlative. For example, it was postulated that STMN1 phosphorylation promoted K562 myeloid leukemia cell proliferation, migration, and invasion since knockdown of PRL-3 mediated these activities [16,17,18]. While STMN1 was phosphorylated on all four serines, the serine (s) which directed these activities was not determined. Together, these diverse differences imply that STMN1 phosphorylation and activities are cell type- and growth factor-specific and that cancer-specific approaches will likely be required to effectively block STMN1 phosphorylation-mediated tumor cell growth and metastasis.

Regrettably, therapies targeting HGF/MET in mCRPC have not been as effective as predicted. Phase I–III clinical trials to block MET activity were performed with cabozatinib (XL-184), a dual MET/VEGFR-2 inhibitor. Cabozatinib appeared promising in a Phase I trial, where 86% of patients with metastatic CRPC showed a significant reduction of bone metastasis and 64% showed a reduction in pain levels [30]. Similar results were obtained in Phase II and III trials; however, both trials were discontinued due to significant adverse side effects [87,88]. In a single-arm Phase II trial using the selective small molecule MET inhibitor AMG337, antitumor activity was observed in 18% of adults with MET-amplified gastric, gastroesophageal junction, and esophageal adenocarcinomas; however, significant adverse side effects were also observed [54]. These trials imply that directly targeting the MET receptor can regulate alternative or non-specific pathways and suggests that inhibiting a selected down-stream target of MET signaling may be more specific with fewer adverse side effects.

Of particular significance, MET and AR expressions are inversely corelated. AR repressed Sp1-induced transcription of MET in a ligand-dependent manner [89], while ADT increased MET levels, implying that the down-regulation of AR activity induced MET which, in turn, promoted PCa cell survival and progression to CRPC [89,90]. In a Phase I study of concurrent enzalutamide and crizotinib administration, improvement in bone pain and reduction in total alkaline phosphatase and plasma serum C-terminal telopeptide (CTX) were not observed; however, significant drug–drug interactions resulted in the active clearance of crizotinib, indicating that suboptimal dosing had occurred [91].

The present investigation underlines the importance of, and the interrelationship between, STMN1, HGF, and MET using publicly available clinical and transcriptomic data of mCRPC and mBC patients. While this analysis was limited by inconsistent annotations of the samples and lack of validation of these clinical observations, this was overcome by analyzing multiple datasets with varying annotations on overall survival, metastatic sites, and treatments. Our study is the first to highlight that HGF/MET signaling phosphorylates STMN1^S16^, and that pSTMN1^S16^ regulates cell cycle progression and proliferation but not metastasis (summarized in Figure 7I). This discovery encourages future investigations into the molecular mechanisms and key signaling pathways by which HGF/MET mediates STMN1-regulated cell growth and metastasis in vitro and in vivo. Furthermore, determining the mechanisms leading to the upregulation of STMN1 and HGF expression in liver metastases may provide insight into the mechanisms by which the liver provides such a favorable microenvironment for the establishment of more aggressive disease. Given that STMN1 relays its multifactorial activities via the phosphorylation of S25, S38, and S63 in addition to S16, determining the impact of HGF/MET signaling on S25, S38, and S63 phosphorylation would provide new knowledge on their roles in promoting proliferation, EMT, and metastatic potential, and whether or not these activities are cancer cell type specific.

## 5. Conclusions

In summary, the STMN1/HGF/MET signaling axis is upregulated in both mCRPC and mBC, modulated by taxane therapy, and STMN1^S16^ phosphorylation regulates cell cycle progression and proliferation but not metastasis. In addition, the expression of STMN1^S16A^ blocks cells in G2/M and promotes apoptosis, mimicking that reported for treatment with docetaxel [92]. Thus, we postulate that modulating STMN1^S16^ phosphorylation status may provide tumor stage-specific windows-of-opportunity to selectively kill tumor cells while preventing/inhibiting the emergence of metastatic disease. While clinical trials have provided proof-of-principle that blocking MET activity reduces bone metastasis and pain levels in mCRPC, decreasing the side effects and adverse pharmacokinetics of drug interactions remain a major challenge. MET remains an important oncogenic driver associated with clonal expansion, metastasis, and cancer cell survival under adverse therapeutic-induced conditions. Thus, developing new approaches for blocking MET kinase-mediated activity is paramount and could include targeting downstream effectors such as STMN1^S16^ phosphorylation to inhibit the cell cycle and tumor cell growth, in addition to more selective small molecule inhibitors or peptides for inhibiting MET, developing alternative strategies such as siRNA-based therapeutics for inhibiting HGF or MET expression, and screening for *MET* mutations to identify new actionable targets. In combination, the inhibition of MET-mediated pSTMN1^S16^ and AR activity could significantly advance the treatment of metastatic cancers.

## Data Availability

cBioPortal (RRID:SCR_014555) was the source of publicly available data on mPCa and mBC biopsy specimens generated by others [36,37]. Data that supports the findings of this study will be made available on request by the corresponding author. Expression microarray data (accession number GSE77930) and primary tumor tissues with clinical annotations and sample sites (Figure 1A–E,G) were downloaded from the Fred Hutchinson Cancer Research Center study on metastatic prostate adenocarcinoma [39]. Clinical annotations, including sample site, castration resistance status, and overall survival data (Figure 1F), were downloaded from the Memorial Sloan Kettering study on metastatic prostate adenocarcinoma [41]. The publicly available ROCplot BC dataset on metastatic breast cancer [44] and ROC Plotter (RIDD:SCR_025347), the ROCplot web-based bioinformatics tool, were used to query for STMN1, HGF, MET, and STM1/MET combined signatures with taxane chemotherapy treatment selected and node positive nodal status filter selected (Figure 1H–N).

## Supplementary Materials

The following supporting information can be downloaded at: https://www.mdpi.com/article/10.3390/cancers17142322/s1, File S1: The original Western Blots; Table S1: Primary antibodies and concentrations used. Table S2: Primers for generating the STMN1 substitution mutations. Figure S1: HGF is expressed in the prostate cancer tumor stroma and not in prostate cancer cells. Figure S2: HGF Concentration differentially modulates DU-145 cell morphology. Figure S3: Oleic acid does not phosphorylate STMN1^S16^. Figure S4: Diagrammatic representation of experimental timelines and serine substitution mutation nomenclature. Figure S5: Flow Cytometry Gating Strategy of synchronized DU145 cells that are treated or transfected at 8 h. Figure S6: STMN1 phosphorylation status HGF/MET signaling do not induce NMuMG and DU-145 cell migration or invasion.

## Abbreviations

The following abbreviations are used in this manuscript:

A	alanine
ADT	androgen deprivation therapy
a.k.a.	also known as
ANOVA	analysis of variance
AR	androgen receptor
BC	breast cancer
BPH	benign prostate hyperplasia
CAMKII	Calcium/Calmodulin-dependent Protein Kinase II
CDK1	cyclin dependent kinase 1
cDNA	copy deoxynucleic acid
CTX	C-terminal telopeptide
E	glutamic acid
ECL	Enhanced Chemiluminescence
EMT	epithelial mesenchymal transition
Fig	figure
FoxM1	Forkhead Box M1
G1/S/G0/M	phases of cell cycle
HGF	hepatocyte growth factor
m	metastatic
mCRPC	metastatic castration-resistant prostate cancer
MET	MET proto-oncogene, Receptor Tyrosine Kinase
p	phospho
p21	Cyclin Dependent Kinase Inhibitor 1A
P53	tumor protein 53
PCa	prostate cancer
pECFP	plasmid enhanced cyan fluorescent protein
pLKO.1/shSTMN1	MISSION^®^ pLKO.1-puro Non-Target shRNA control
RNA	ribonucleic acid
STMN1	Stathmin 1
S	serine
sh	Short hairpin
Sp1	Sp1 transcription factor
t	total
VEGFR-2	vascular endothelial growth factor receptor 2
