# Supplementary material for: Stathmin Serine 16 Phosphorylation Is a Key Regulator of Cell Cycle Progression Without Activating Migration and Invasion In Vitro
Source: Cancers (Basel). 2025 Jul 12;17(14):2322. doi: 10.3390/cancers17142322 (PMC12293763; doi:10.3390/cancers17142322)
Supplement: Supplementary file 1 [file cancers-17-02322-s001.zip › Deford et al_Supplementary_tables_2025-05-20_MDPI.pdf]

Stathmin Serine 16 Phosphorylation is a Key Regulator of Cell Cycle Progression Without Activating Metastatic Behavior

Paul L. Deford[1*], Andrew P. VonHandorf[2*], Brian G. Hunt[3], Simran Venkatraman[1], Susan E. Waltz[3], Katherine A. Burns[1], and Susan Kasper[1]

1 Department of Environmental and Public Health Sciences, University of Cincinnati College of Medicine, Kettering Laboratory, 160 Panzeca Way, Cincinnati, OH 45267-0056, USA
2 Center for Autoimmune Genomics and Etiology, Cincinnati Children's Hospital Medical Center 3333 Burnet Avenue Cincinnati, OH 45229-3039, USA
3 Department of Cancer Biology, University of Cincinnati College of Medicine, Vontz Center for Molecular Studies, 3125 Eden Avenue, PO Box 670521, Cincinnati, OH 45267-0521, USA

Supplemental tables 1 and 2

Table S1. Primary antibodies and concentrations used.

Antibody	Target Protein	Company	Product Number/Code	RRID	Conjugate	Dilution
Cyclin A2 (BF683) Mouse mAb	Cyclin A2	Cell Signaling	4656	AB_2071958	None	1:1000
Cyclin B1 (V152) Mouse mAb	Cyclin B1	Cell Signaling	4135	AB_2233956	None	1:1000
cyclin D1 (A-12) Mouse mAb	Cyclin D1	Santa Cruz	8396	AB_627344	None	1:1000
anti-Cyclin E1 (HE12) Mouse mAb	Cyclin E1	Cell Signaling	4129	AB_2071200	None	1:1000
GAPDH Mouse mAb	GAPDH	Proteintech	60004-1-lg	AB_2107436	None	1:2000
HGF β (D6S7D) XP® Rabbit mAb	HGF	Cell Signaling	52445	AB_2799412	None	1:1000
Met (D1C2) XP Rabbit mAb	Met	Cell Signaling	8198	AB_10858224	None	1:1000
Phospho-Met (Tyr1234/1235) (D26) XP® Rabbit mAb	pMET	Cell Signaling	3077	AB_2143884	None	1:750
p21 Waf1/Cip1 (12D1) Rabbit mAb	p21	Cell Signaling	2947	AB_823586	None	1:1000
Op18 (E-3) Mouse mAb	STMN1	Santa Cruz	55531	AB_630260	None	1:1000
Phospho-Stathmin 1 (Ser16) Rabbit Antibody (Clonality unknown)	pSTMN1 Ser16	Thermo Fisher / Invitrogen	PA5-17091	AB_10979092	None	1:250

Table S2. Primers for generating the STMN1 substitution mutations.

Serine (S)	Codon	Substitution	Alanine (A)	Primers for S>A Substitution	bp
16	TCA	c.46T>G	**g**CA	(f) 5'-ggagaagcgtgccgcaggccaggcttt-3'<br>(r) 5'-aaagcctggcctgcggcacgcttctcc-3'	27
25	AGC	c.73A>G<br>c.74G>C	**gc**C	(f) 5'-ggcttttgagctgattctcgcccctcggtcaaaagaatct-3'<br>(r) 5'-agattcttttgaccgaggggcgagaatcagctcaaaagcc-3'	40
38	TCC	c.112T>G	**g**CC	(f) 5'-cttctttggaggggcaagggggaattctggaacag-3'<br>(r) 5'-ctgttccagaattcccccttgcccctccaaagaag-3'	35
63	TCC	c.187T>G	**g**CC	(f) 5'-cagaagaaagacgcaaggcccatgaagctgaggtc-3'<br>(r) 5'-gacctcagcttcatgggccttgcgtctttcttctg-3'	35
**Serine (S)**	**Codon**	**Substitution**	**Glutamic Acid (E)**	**Primers for S>E substitution**	**bp**
16	TCA	c.46T>G<br>c.47C>A	**ga**A	(f) 5'-actggagaagcgtgccgaaggccaggcttttgag-3'<br>(r) 5'-ctcaaaagcctggccttcggcacgcttctccagt-3'	34
25	AGC	c.73A>G<br>c.74G>A<br>c.75C>G	**gag**	(f) 5'-caggcttttgagctgattctcgagcctcggtcaaaagaatctgtt-3'<br>(r) 5'-aacagattcttttgaccgaggctcgagaatcagctcaaaagcctg-3'	45
38	TCC	c.112T>G<br>c.113C>A<br>c.114C>A	**gaa**	(f) 5'-gaatctgttccagaattcccccttgaacctccaaagaagaaggatcttt-3'<br>(r) 5'-aaagatccttcttctttggaggttcaagggggaattctggaacagattc-3'	49
63	TCC	c.187T>G<br>c.188C>A<br>c.189C>A	**gaa**	(f) 5'-gctgcagaagaaagacgcaaggaacatgaagctgaggtcttgaag-3'<br>(r) 5'-cttcaagacctcagcttcatgttccttgcgtctttcttctgcagc-3'	45

Human STMN1 Reference sequence: pECFP-N1. STMN1 (Addgene, plasmid #86783; RRID: Addgene_86783)
