# Supplementary material for: Stathmin Serine 16 Phosphorylation Is a Key Regulator of Cell Cycle Progression Without Activating Migration and Invasion In Vitro
Source: Cancers (Basel). 2025 Jul 12;17(14):2322. doi: 10.3390/cancers17142322 (PMC12293763; doi:10.3390/cancers17142322)
Supplement: Supplementary file 1 [file cancers-17-02322-s001.zip › Deford et al_Supporting Information_figures_2025-07-11.pdf]

Stathmin Serine 16 Phosphorylation is a Key Regulator of Cell Cycle Progression Without Activating Metastatic Behavior

Paul L. Deford[1*], Andrew P. VonHandorf[2*], Brian G. Hunt[3], Simran Venkatraman[1], Susan E. Waltz[3], Katherine A. Burns[1], and Susan Kasper[1]

1 Department of Environmental and Public Health Sciences, University of Cincinnati College of Medicine, Kettering Laboratory, 160 Panzeca Way, Cincinnati, OH 45267-0056, USA
2 Center for Autoimmune Genomics and Etiology, Cincinnati Children's Hospital Medical Center 3333 Burnet Avenue Cincinnati, OH 45229-3039, USA
3 Department of Cancer Biology, University of Cincinnati College of Medicine, Vontz Center for Molecular Studies, 3125 Eden Avenue, PO Box 670521, Cincinnati, OH 45267-0521, USA

Supplemental figures 1 to 5 and legends

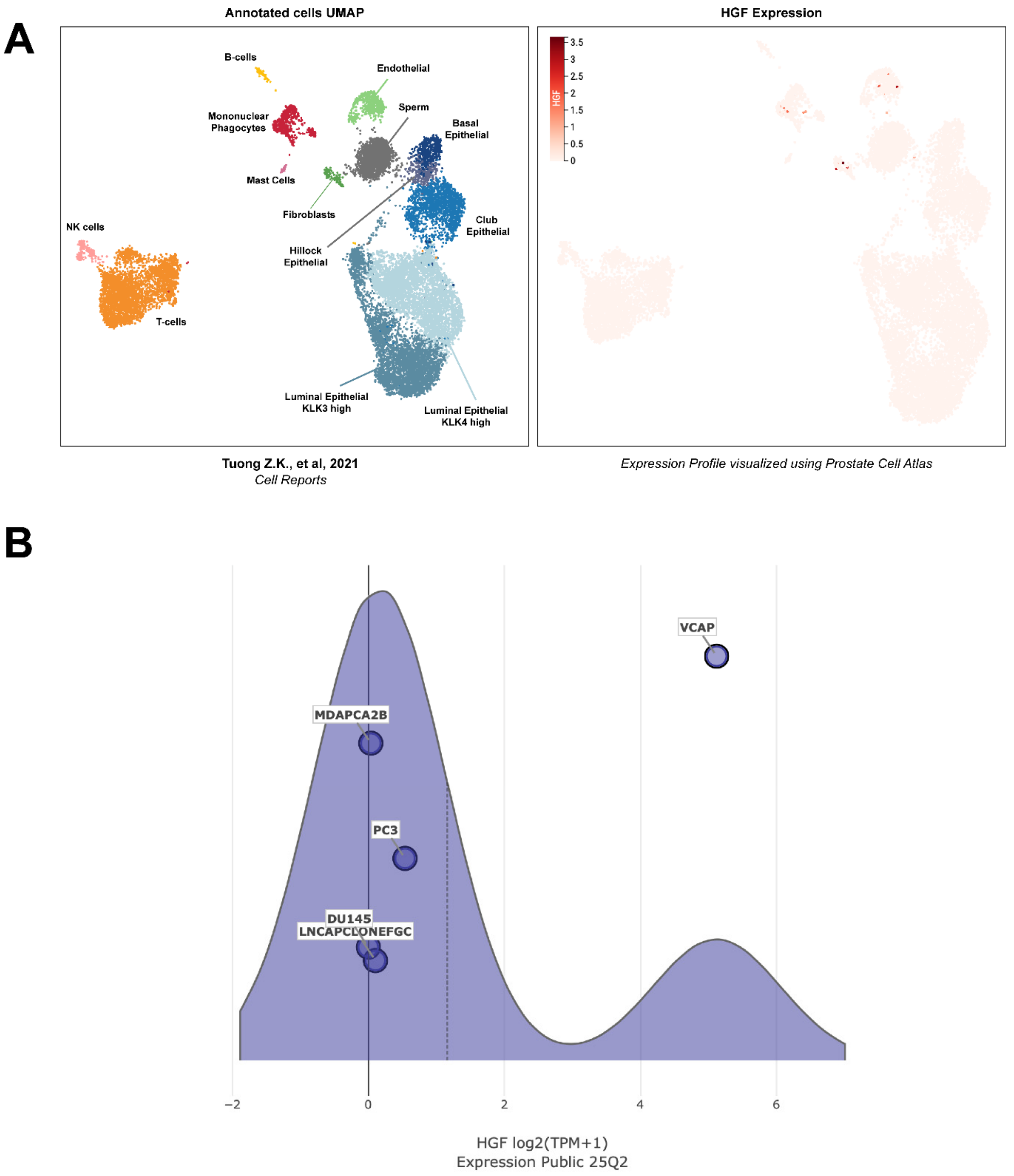

Figure S1: HGF is expressed in the prostate cancer tumor stroma and not in prostate cancer cells. A. Single-cell RNA sequencing data of 10 paired prostate cancer tissues and adjacent normal tissues was analyzed for HGF expression using the Prostate Cell Atlas portal (prostate_portal_300921). Cell had been clustered and annotated as per Tuong et al's publication. **B.** Cancer Cell Line Encyclopedia transcriptomic dataset was analyzed for HGF expression using the DepMap portal. Cell lines were filtered by the Disease Subtype, 'Prostate Adenocarcinoma'.

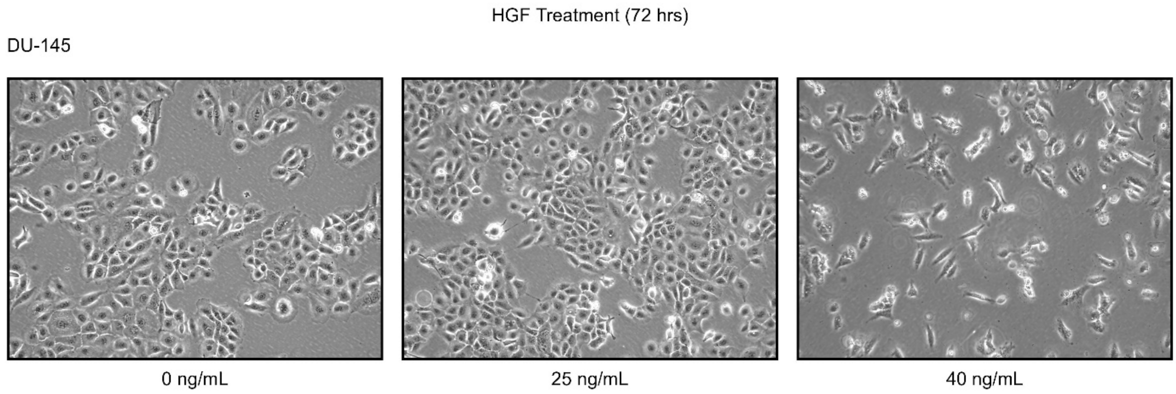

Figure S2. HGF concentration differentially modulates DU-145 cell morphology. DU-145 cells were treated with 0, 25 and 40 ng/mL HGF for 72 h. Phase contrast; magnification, 10x.
In asynchronized DU-145 cells, cells treated with 25 ng/mL maintained a classic cobblestone-like morphology. In contrast, cells treated with 40 ng/mL HGF exhibited a spindle shape-like morphology reminiscent of the scatter factor properties of HGF (26). Therefore, 25 ng/mL HGF was used in subsequent experiments to ensure that cells exhibited a cobble-stone morphology and typical DU-145 proliferation and contact inhibition characteristics. Additional information is provided in the text of the manuscript.

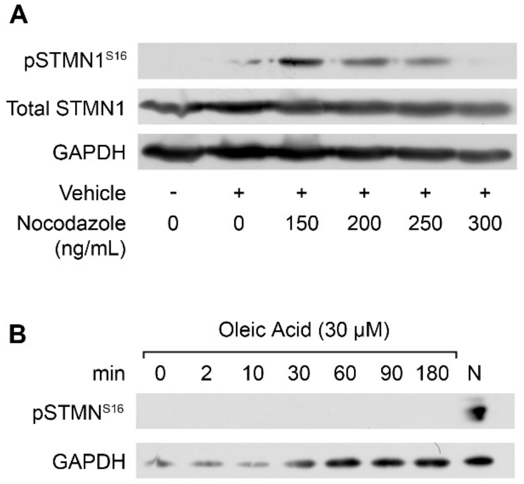

Figure S3. Oleic acid does not phosphorylate STMN1^{S16}. **A**. Nocodazole is an agent used by Thermo Fisher Scientific (and other companies) to phosphorylate S16 and validate their Phospho-Stathmin (Ser16) Antibody # PA5-17091. Treatment of DU-145 cells with increasing concentrations of Nocodazole determined that 150 ng/ml Nocodazole was optimal for phosphorylating STMN1^{S16}. **B**. Thus, 150 ng/ml Nocodazole was used as a positive control to determine whether oleic acid could phosphorylate STMN1^{S16}. CaMKII activity does not phosphorylate pSTMN1^{S16}. Cells were treated with 30μM oleic acid over time as indicated, followed by western blot analysis. Nocodazole (150 ng/ml) served as a positive control for pSTMN1^{S16}.

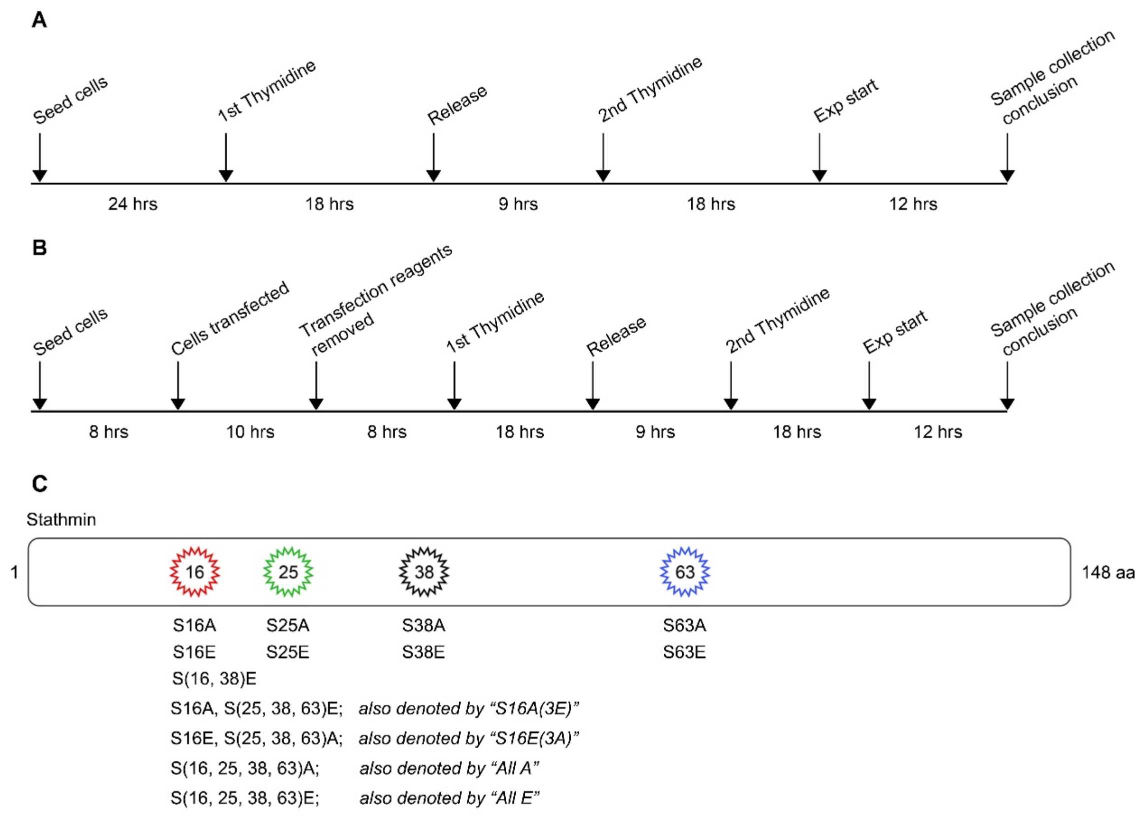

Figure S4. Diagrammatic representations of experimental timelines and serine substitution mutation nomenclature. Timelines for establishing the double thymidine block in **(a),** non-transfected cells and **(b)**, transfected cells. **(c)**. Position of the 4 serines phosphorylated in STMN1 and a summary of the serine substitution mutations generated and analyzed in DU-145 and/or NMuMG cells. Additional information for generating the S to A and S to E substitution mutations is provided in Table S2.

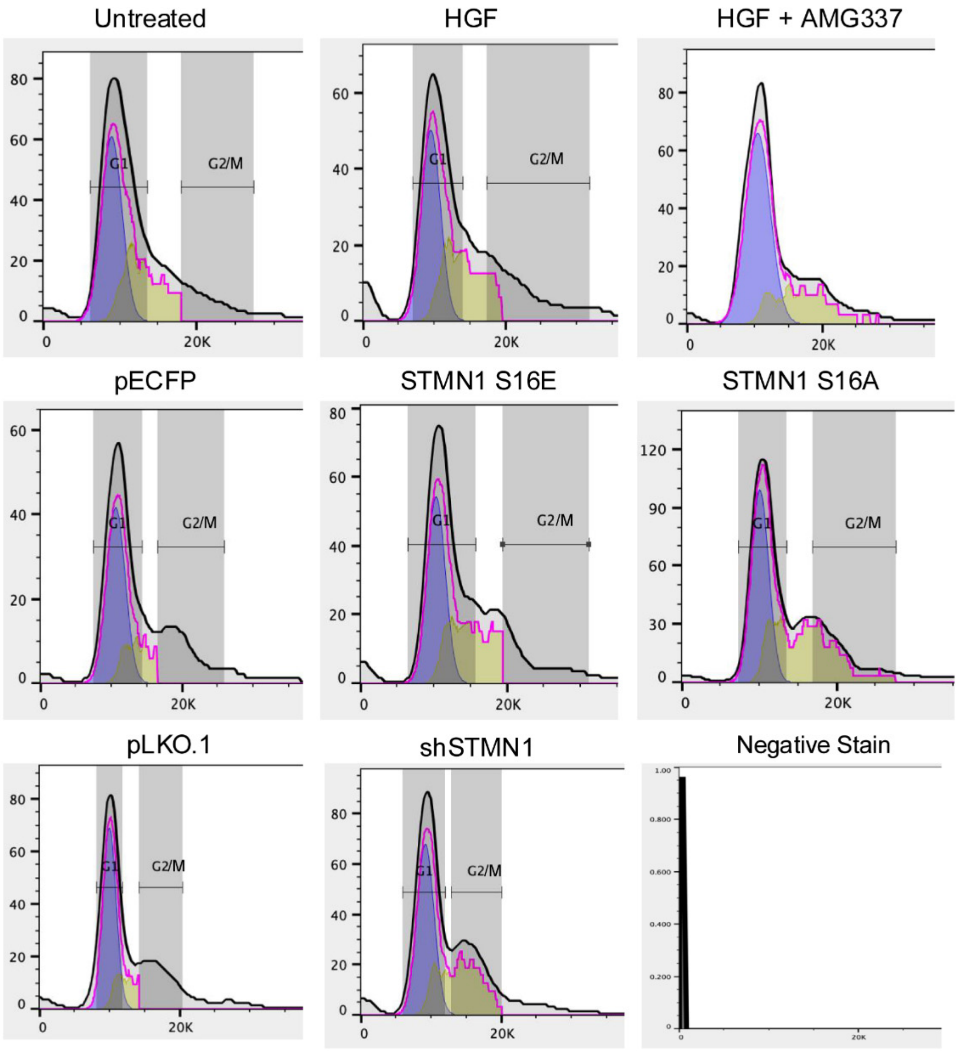

Figure S5. Flow Cytometry Gating Strategy at 8 hours for DU145 cells either treated with or without HGF and MET inhibitor AMG337 or were transfected with the pECFP empty vector control, or with pECFP expressing wild type (wt) STMN1, or S16, S25 substitution mutants where S was substituted with alanine (A) or glutamic acid (E) to mimic dephosphorylation and phosphorylation, and pLKO.1 control (containing scrambled RNA), pLKO.1/shSTMN1.

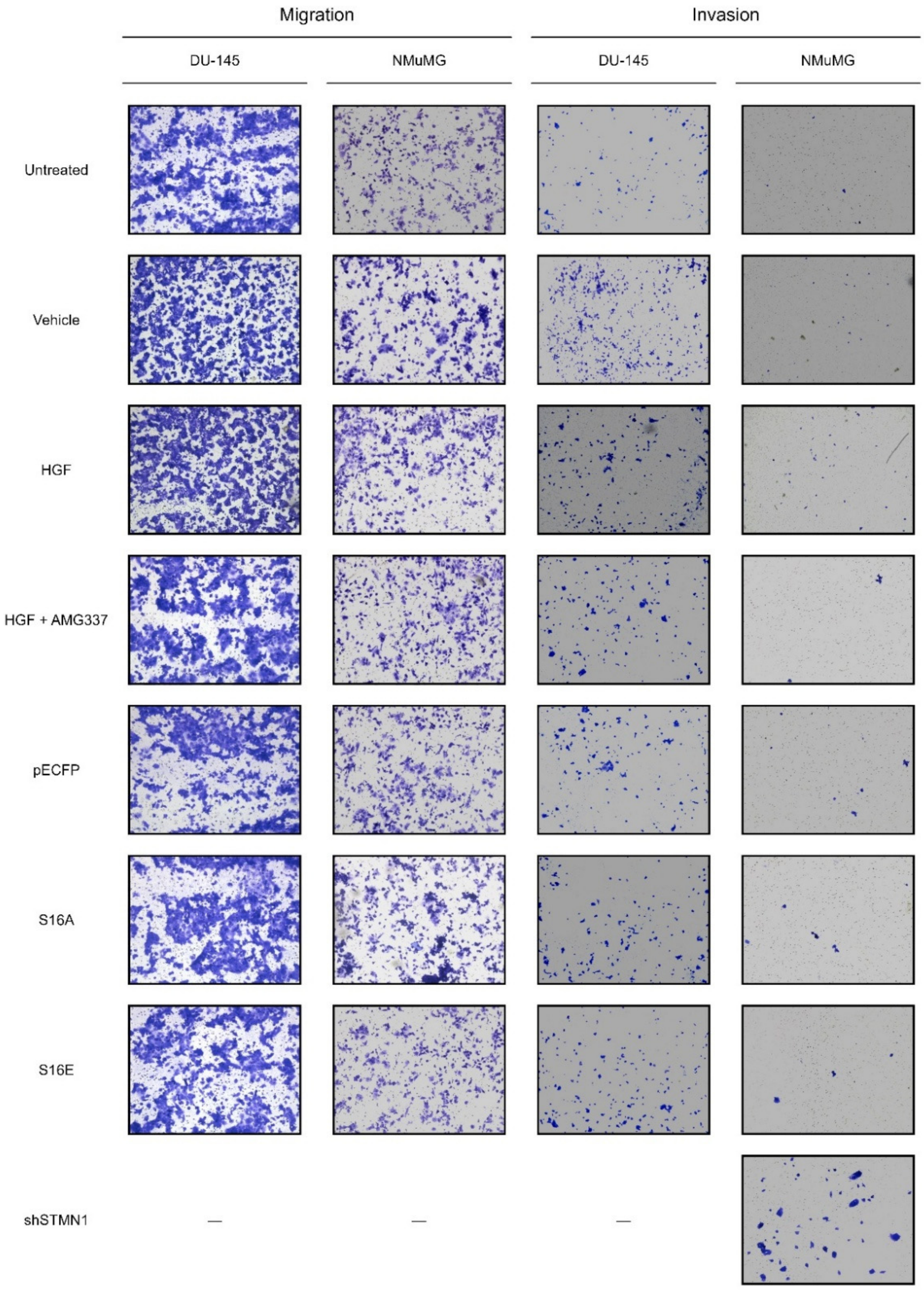

Figure S6. STMN1 phosphorylation status and HGF/MET signaling do not induce NMuMG and DU-145 cell migration or invasion. Images of migrating cells (non-coated membranes) and invading cells (Matrigel-coated membranes) are provided for NMuMG and DU-145 cells as indicated. shSTMN1 served as a positive metastasis control for NMuMG cells which exhibit very low invasive properties compared to DU-145 cells (10). Analysis of the migration and invasion data are presented in **Fig. 7C**.
