# Supplementary figures and images for: Stathmin Serine 16 Phosphorylation Is a Key Regulator of Cell Cycle Progression Without Activating Migration and Invasion In Vitro
Source: Cancers (Basel). 2025 Jul 12;17(14):2322. doi: 10.3390/cancers17142322 (PMC12293763; doi:10.3390/cancers17142322)

Figure 2C

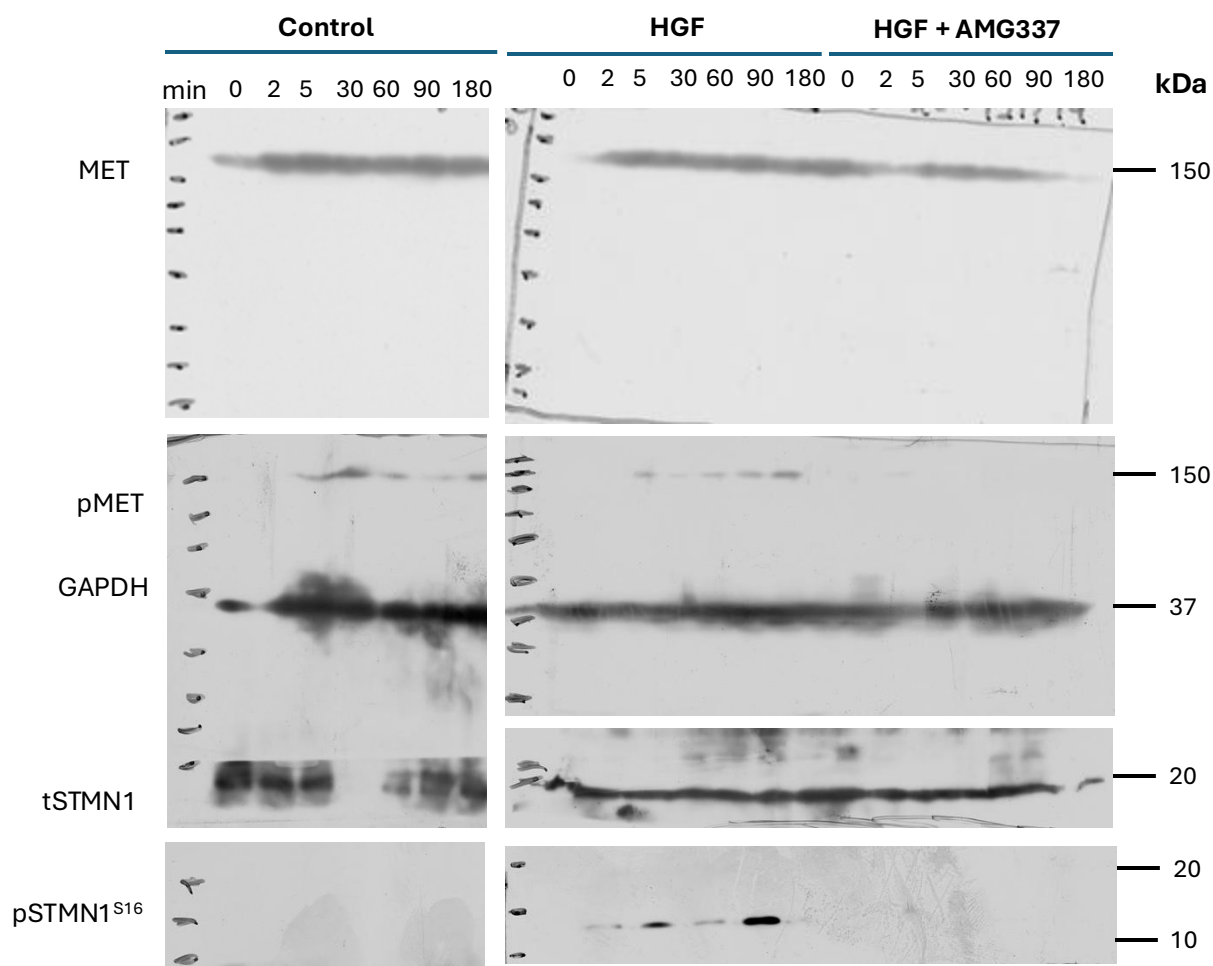

Figure 3

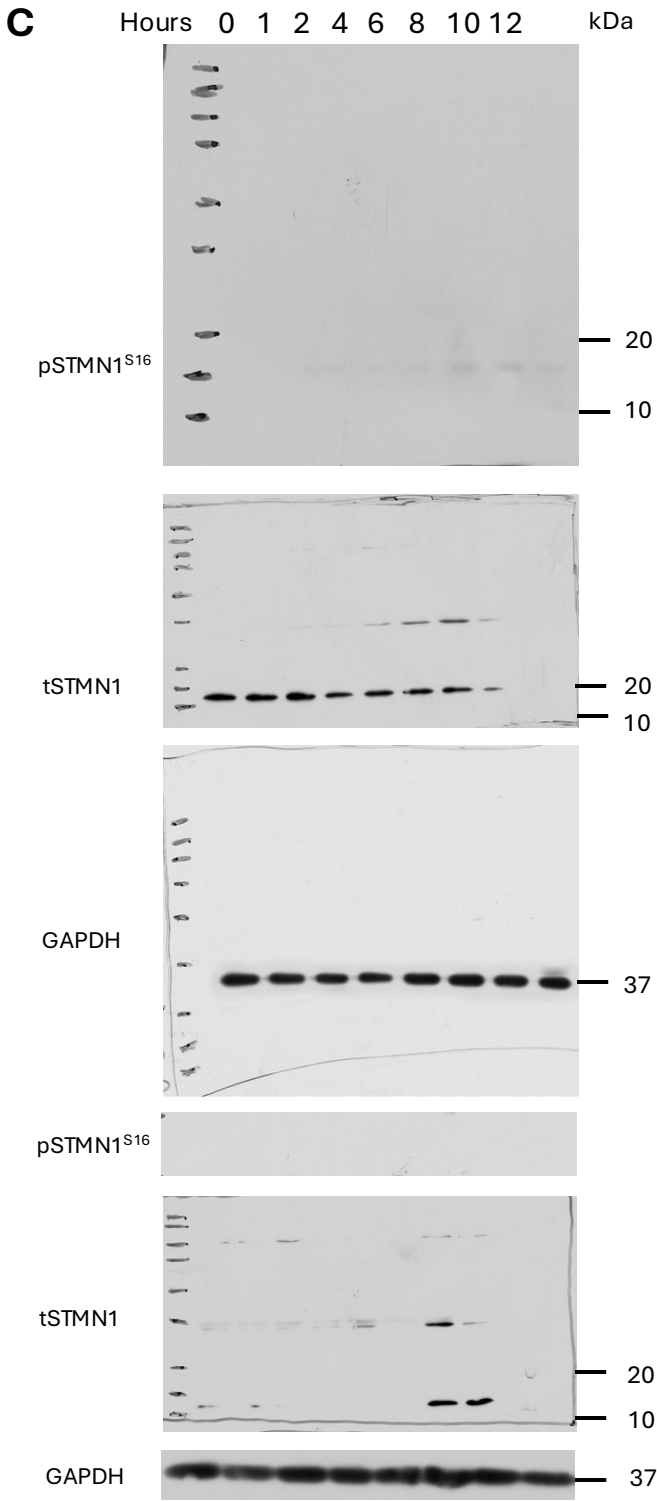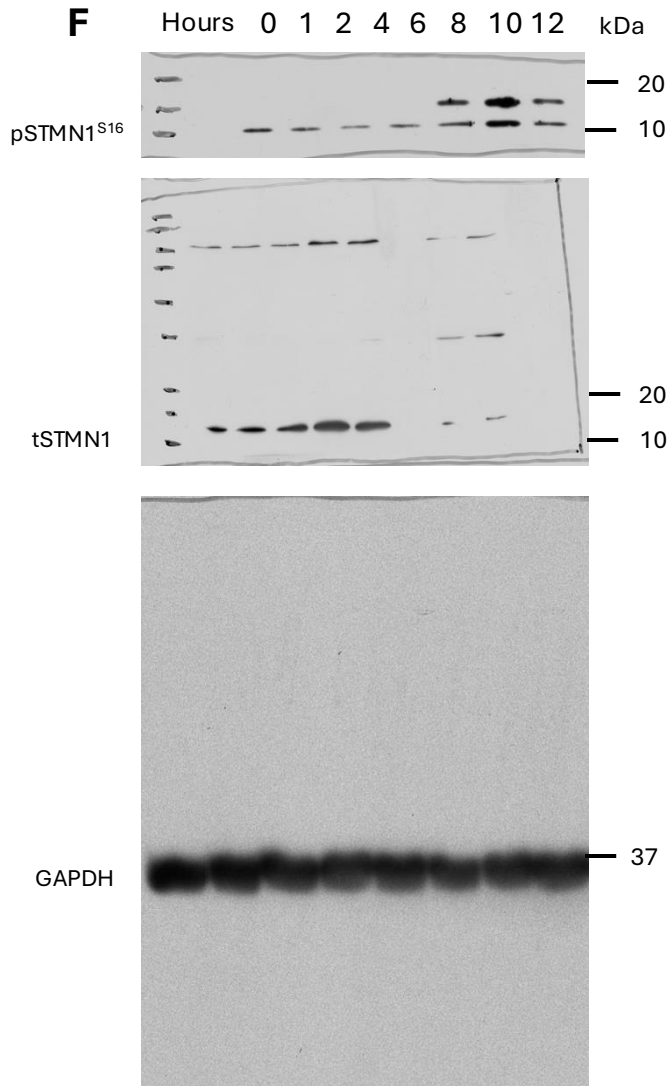

**Figure 4**

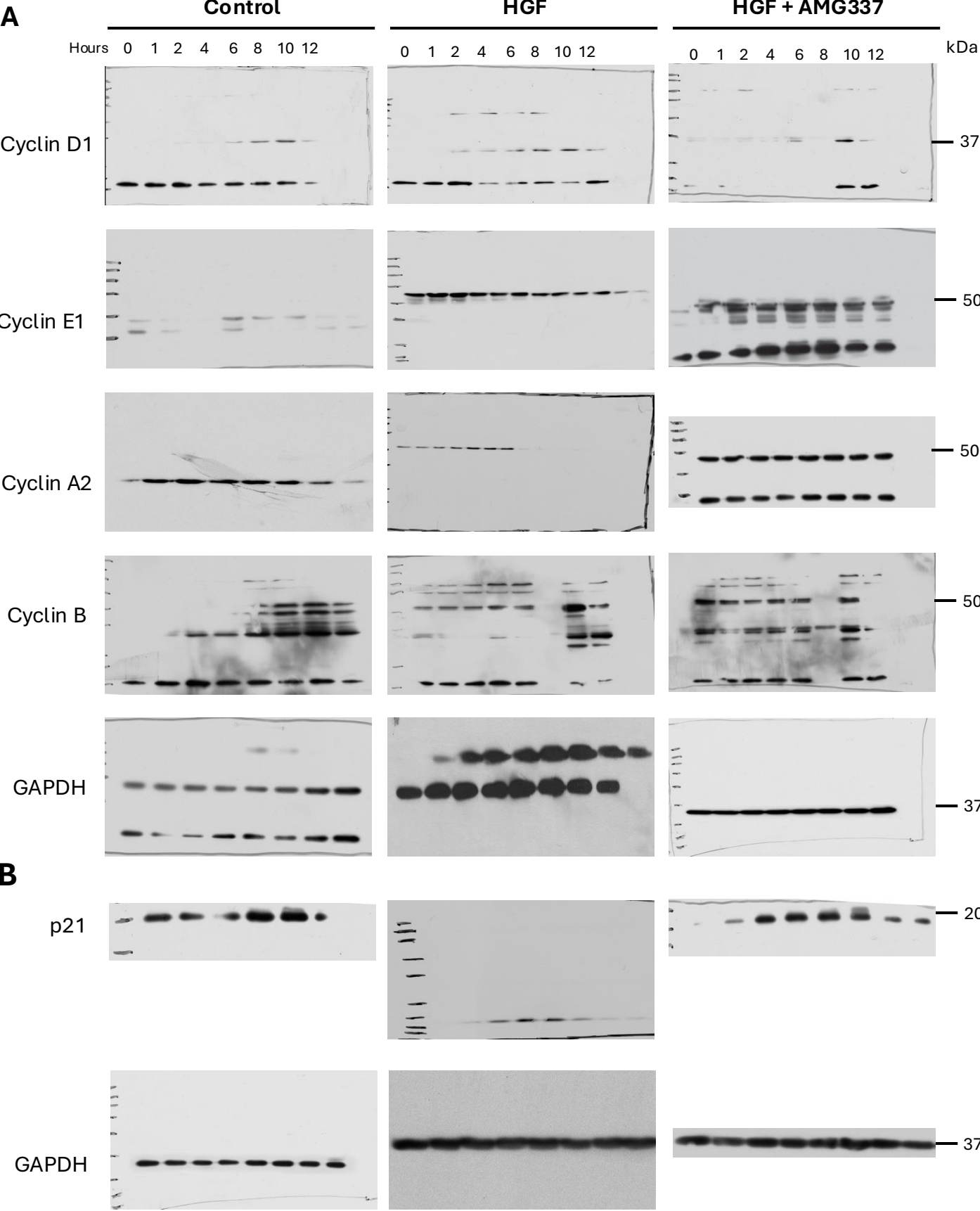

Figure 6

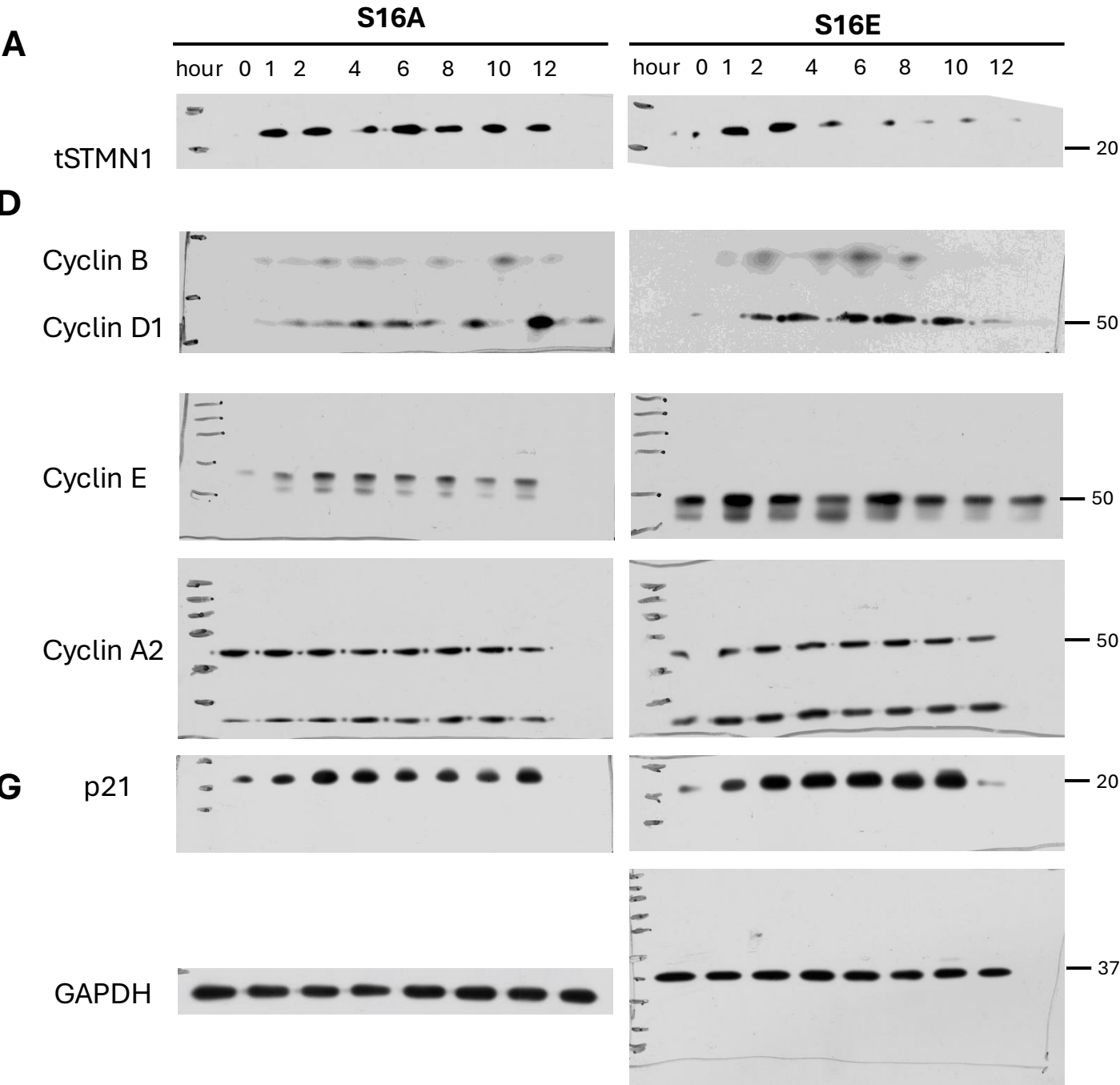

Figure S3

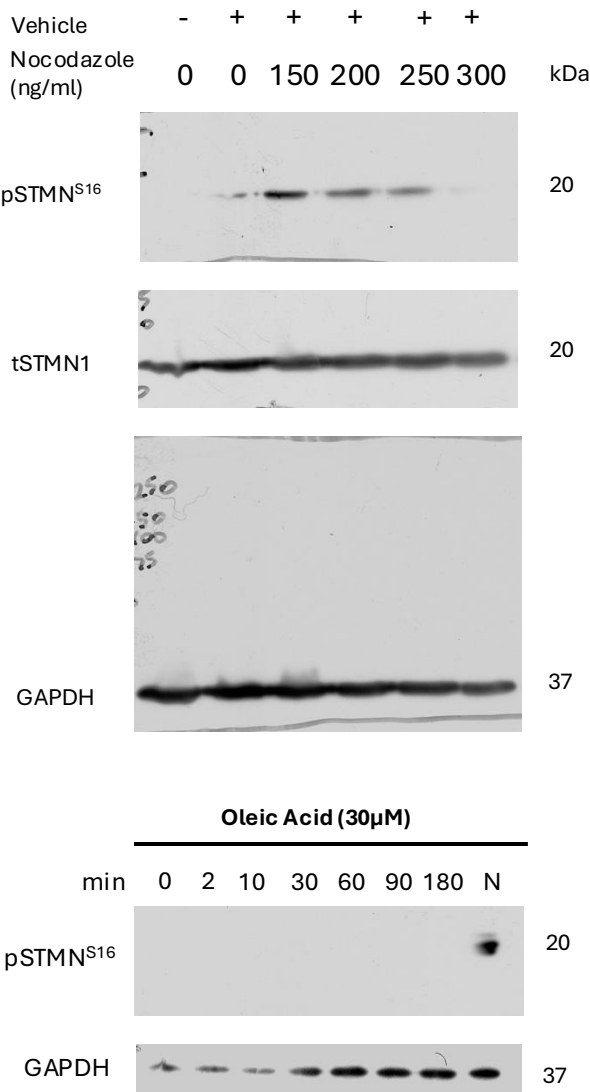

Supplement: Supplementary file 1 [file cancers-17-02322-s001.zip › File S1. Western Blots revised_proof.pdf]
